# Supplementary material for: Local shape descriptors for neuron segmentation
Source: Nat Methods. 2022 Dec 30;20(2):295–303. doi: 10.1038/s41592-022-01711-z (PMC9911350; doi:10.1038/s41592-022-01711-z)
Supplement: Supplementary file 1 — Supplementary Note, Supplementary Figs. 1–16, Supplementary Tables 1–11. [file 41592_2022_1711_MOESM1_ESM.pdf]

nature methods

Article

https://doi.org/10.1038/s41592-022-01711-z

Local shape descriptors for neuron segmentation

In the format provided by the authors and unedited

Supplementary Note

Contents

A Main text 1
- A.1 Introduction . . . 1
  - A.1.1 Neuron Segmentation Methods . 1
  - A.1.2 Contributions . . . 2
- A.2 Results . . . 2
  - A.2.1 Metrics for Neuron Segmentation 2
  - A.2.2 Datasets . . . 3
  - A.2.3 Neuron Segmentation Accuracy . 3
- A.3 Discussion . . . 4
  - A.3.1 Metric Evaluation . . . 4
  - A.3.2 Auxiliary Learning for Boundary Prediction . . . 4
  - A.3.3 Auto-Context Refinement . . . 5
  - A.3.4 Masking . . . 5
- A.4 Conclusions and Future Directions . . . 5

B Local Shape Descriptors 5

C Min-Cut Metric 6

D Zebrafinch 6
- D.1 Training . . . 6
  - D.1.1 Data . . . 6
  - D.1.2 Networks . . . 6
  - D.1.3 Pipeline . . . 7
- D.2 Prediction . . . 7
- D.3 Segmentation . . . 7
  - D.3.1 Watershed . . . 7
  - D.3.2 Agglomeration . . . 7
  - D.3.3 Segment . . . 7
- D.4 Evaluation . . . 7

E FIB-SEM volumes 8
- E.1 Training . . . 8
  - E.1.1 Data . . . 8
  - E.1.2 Networks . . . 8
  - E.1.3 Pipeline . . . 8
- E.2 Prediction . . . 8
- E.3 Segmentation . . . 8
  - E.3.1 Watershed . . . 8
  - E.3.2 Agglomeration . . . 8
  - E.3.3 Segment . . . 8
- E.4 Evaluation . . . 8

F Throughput 9

G Extended Experiments 9
- G.1 Serial Section Data (ssTEM) . . . 9
- G.2 Ablations . . . 9
- G.3 Non-EM Data: Epithelial Cells . . . 9

A Main text

A.1 Introduction

A.1.1 Neuron Segmentation Methods

Neuron reconstruction is an instance segmentation problem. Unlike semantic segmentation, in which the goal is to assign every voxel to a specific class, instance segmentation assigns all voxels belonging to the same object a unique label. Since those labels can not be predicted directly, alternative local representations are sought, which permit extraction of globally unique labels in a subsequent processing step.

The most straight forward local representation is to label pixels as either foreground or background, and then perform a connected component analysis limited to foreground pixels to extract unique objects. However, in the case of 3D neuron segmentation this approach often fails to distinguish voxels in finer neurites, where the axial resolution of the data is lower (Ciresan et al., 2012). To deal with those situations, several methods have centered around the prediction of affinities (*i.e.*, the labeling of edges between neighboring voxels as "connected" or "cut"), rather than labeling the voxels themselves (Turaga et al., 2010; Funke et al., 2019).

Affinities effectively increase the resolution of the prediction, but otherwise inherit the advantages and disadvantages of voxel-wise boundary labeling: Both can be computed locally during training and inference, which allows for trivial parallelization. However, segmentations extracted from those predictions are sensitive to small errors: A few incorrectly assigned voxels (or edges between voxels) can label a boundary as foreground, resulting in two segments becoming falsely merged during post-processing.

Those so-called merge errors are the notorious failure modes of neuron segmentation methods. Merge errors have generally been considered worse than split errors, since they have the potential to propagate throughout a dataset. Even if just two neurons are merged together, the resulting segmentation can be difficult to resolve for proofreaders, if the neurons in question have several contact sites. This has been a particular concern for the first generation of proof-reading tools, which did not provide algorithmic help to split wrongly merged objects. In those situations, the origin point of a false merge would first need to be found before the objects can be separated; a task akin to searching for a needle in a haystack.

To avoid those merge errors, Briggman et al. (2009) introduced Malis, a loss function that penalizes topological errors by minimizing the Rand Index. The Rand Index naturally favors split errors over merge errors and thus helped to bias boundary predictions to split instead of merge in ambiguous situations. Funke et al. (2019) expanded this method by constraining the loss to a positive and negative pass, and by providing a maximal spanning tree formulation of the loss, which allows for a quasi-linear and exact computation of the loss during training.

More recent methods do not explicitly focus on merge errors, which is a possible consequence of improved proofreading tools that allow users to separate objects with just a few interactions. Lee et al. (2017) found that using an increased affinity neighborhood acts as an auxiliary learning objective to improve direct neighbor affinities. This work demonstrates that auxiliary learning helps to make better use of local context in the receptive field of the neural network. The nature of this auxiliary learning approach is similar to the LSDs proposed here.

All affinity-based methods have in common that they need a subsequent agglomeration step to produce a final segmentation. Methods such as watershed variants (Wolf et al., 2018) and constrained agglomeration (Beier et al., 2017) successfully demonstrated an increase in robustness of the resulting segmentation to small errors in the predicted affinities.

Not all neuron segmentation methods are based on boundary predictions. The most notable exception are Flood-Filling Networks (FFN), the current state of the art in terms of segmentation quality (Januszewski et al., 2018). FFN eliminated the need for a

multi-step segmentation process by using a recurrent convolutional neural network to fill neurons iteratively in an end-to-end fashion. Given seed points within neurons, the algorithm predicts which voxels belong to the same object as the seeds. This approach has been proven to be successful on very large volumes, although it is computationally more expensive than its affinity-based counterparts.

More recently, another promising alternative to boundary prediction has been proposed by Lee et al. (2021), which uses metric learning to produce dense voxel embeddings. The embeddings of voxels that belong to the same object are encouraged to be close in embedding space, while the embeddings of voxels of different objects are pushed away from each other. Clustering of the embeddings then reveals a segmentation. Since object similarity or dissimilarity can only be discerned locally, the method is applied in a block-wise fashion and the segmentations of neighboring blocks are stitched together to process a whole volume.

A.1.2 Contributions

We introduce LSDs as an auxiliary learning task for affinity predictions and demonstrate that segmentation results are competitive with the current state of the art, albeit two orders of magnitude more efficient to compute. LSDs are 10-dimensional vectors, computed for each voxel, which encode local object properties. We engineered LSDs to describe features that could be leveraged to improve boundary detection. Specifically, they consist of three parts: the local size of the object (1D), the offset to the local center of mass (3D), and the local directionality (6D) (described in detail in Methods and Supplementary Section B).

We conducted a large comparative study of recent neuron segmentation algorithms. Specifically, we evaluated four of the aforementioned methods against three LSD-based methods on the following datasets:

- **Zebrafinch:** A region consisting of ~10^6µm^3 (~663 gigavoxels) of songbird neural tissue, imaged using serial block-face EM at 9x9x20 nanometer (xyz) resolution (Januszewski et al., 2018). 0.02% of the full dataset was used to train networks (dense segmentations), 12 manually traced neuron skeletons (13.5 mm) were used for validation and 50 skeletons (97 mm) were used for testing.
- **Hemi-brain:** Three volumes containing ~1650µm^3, ~4750µm^3, and ~10360µm^3 (~33 total gigavoxels) of raw data, cropped from the ~26 teravoxel dataset generated by Scheffer et al. (2020). This volume was taken from the central brain of a *Drosophila* and imaged with FIB-SEM at 8 nanometer isotropic resolution. 0.002% of the data was used for training (dense segmentation), and 0.06% for testing using a whitelist of proofread neurons.
- **Fib-25:** A ~1.8×10^5µm^3 (~346 gigavoxels) volume from the *Drosophila* visual system was imaged with FIB-SEM at 8 nm isotropic resolution (Takemura et al., 2015). 0.09% of the data was used for training (dense segmentation). Testing was restricted to a 13.8 gigavoxel region using a whitelist of proofread neurons.

For each dataset, we compare LSD-based methods against three previous affinity-based methods: (1) direct neighbor and (2) long-range affinities with mean squared error (MSE) loss, and (3) direct neighbor affinities with Malis loss. Each affinity-based method (including our LSD methods) was trained in the same way and uses the same network architecture (where possible). We used the same segmentation extraction method (from Funke et al. (2019)) to convert the predicted affinities into segmentations.

We further include a comparison against FFN segmentations, which were made available to us by the authors of Januszewski et al. (2018).

We make the training scripts and datasets used in this study publicly available in a central repository[1], in the hope that non-affinity-based methods that we did not cover in this study (like the recent deep metric learning proposed in Lee et al. (2021)) can be compared in a similar manner.

We compare the aforementioned methods against three different architectures that use LSDs as an auxiliary loss: a simple multitask approach (MtLsd) and two auto-context approaches (AcLsd and AcRLsd). We summarize those methods briefly in the following, for a detailed description see Methods.

MtLsd uses a similar strategy to the long-range affinity neighborhood proposed by Lee et al. (2017). The network is taught to simultaneously predict LSDs and affinities (Figure 1.E).

AcLsd and AcRLsd use an auto-context learning strategy, as proposed by Tu and Bai (2010). This strategy attempts to refine the quality of a prediction by using a cascade of predictors. For voxel classification, for example, the first pass of an auto-context classifier predicts voxel labels from raw data. The second pass then uses those predictions from the first pass as input[2]. We loosely adapted this idea when designing our auto-context networks. We first taught a network to predict LSDs from raw EM data. The predicted LSDs were then passed into a second network in order to learn affinities (Figure 1.E).

We generally observe an increase in affinity prediction accuracy when training to predict LSDs in an auxiliary task. This increase is most noticeable when using an auto-context setup. We evaluated all methods using two commonly used neuron segmentation accuracy metrics, Variation of Information (VOI) and Expected Run-Length (ERL). We generally find on-par performance between LSD-based methods and FFN under VOI, but inferior scores for LSD-based methods under ERL.

We further investigated how VOI and ERL relate to proof-reading effort given the capabilities of contemporary proof-reading tools (Zhao et al., 2018; Dorkenwald et al., 2022) is a considerably more reliable proxy for proof-reading effort than ERL (see Figure 4, Supplementary Figure 9, and Discussion). To this end, we developed a novel metric, the Min-Cut Metric (MCM), to count the number of split and merge operations needed to correct an automatic segmentation. We find general ranking agreement between VOI and MCM, but not between ERL and MCM, which likely stems from ERL's sensitivity to merge errors. Our results suggest that VOI can serve as a proxy to measure proof-reading effort.

A.2 Results

A.2.1 Metrics for Neuron Segmentation

A.2.1.1 Variation of Information (VoI)

A metric to compare clusterings (Meilă, 2007), which became an established metric to assess neuron segmentation accuracy. VoI measures the disagreement between two segmentations in terms of the average number of bits needed to guess the segment ID of a

[1]https://github.com/funkelab/lsd

[2]See https://www.ilastik.org/documentation/autocontext/autocontext for a popular example of this strategy.

randomly chosen voxel in one segmentation, given only its label in the other segmentation. This measurement is performed in both directions, giving rise to the two additive components of VoI, a measure for split and merge errors. Lower values are better, with equivalent segmentations (up to label permutations) having a value of zero.

A.2.1.2 Expected Run-Length (ERL)

Following the assumption that false merges are in practice harder to correct than false splits, Januszewski et al. (2018) proposed to measure accuracy in terms of the Expected Run Length (ERL), which measures the expected length of an error-free path along neurons in a volume. Notably, all paths contained in falsely merged segments are considered erroneous, thus ERL emphasizes merge errors disproportionally. An appealing aspect of ERL is that it relates segmentation errors to cable length, a commonly used and interpretable feature of neurons.

A.2.1.3 Min-Cut Metric (MCM)

A metric that assumes that a user can directly interact with agglomerated fragments from an oversegmentation. In particular, we assume that users can split segments by means of a min-cut through the fragment graph between two selected fragments, where edge costs correspond to the merge scores used during agglomeration. In this context, the MCM reports the number of split and merge operations needed to be performed by a human annotator to obtain the desired segmentation.

Notably, a merge of two neurons can be resolved with a single interaction, even if the neurons have several contact sites. The min-cut solution will identify all necessary cuts to separate the two selected fragments. For most commonly used agglomeration algorithms (and the one used here), only one cut is necessary, as connectivity on the fragment graph is defined by a single linkage clustering (Funke et al., 2019).

This metric is of practical relevance since min-cuts on fragment graphs are used to split merged objects in commonly used proof-reading tools (Zhao et al., 2018; Dorkenwald et al., 2022). The details of this metric are described in Supplementary Section C.

A.2.2 Datasets

A.2.2.1 Hemi-Brain

The so-called HEMI-BRAIN is a FIB-SEM volume of the *Drosophila melanogaster* central brain, imaged at 8nm isotropic resolution (Scheffer et al., 2020), comprising a total of 26 teravoxels of image data. We evaluate all investigated methods on regions restricted to the ELLIPSOID BODY, a neuropil implicated in spatial navigation, (Turner-Evans and Jayaraman, 2016), which contained ample ground-truth data for evaluation.

We used eight volumes of densely annotated ground-truth volumes containing ~450µm^3 of labeled data for training. Three RoIs with ~12µm, ~17µm, and ~22µm edge lengths were cropped from the larger volume, and prediction was done directly on each RoI.

Supervoxels were limited to the ELLIPSOID BODY using a mask[3] and then agglomerated using the same two merge functions as in the ZEBRAFINCH dataset.

[3]Kindly provided by the Janelia FlyEM project team (https://janelia.org/project-team/flyem)

We produced segmentations for each network over a range of thresholds on the RoIs, and consolidated a single FFN segmentation[7]. A densely labeled ground-truth volume was cropped and filtered using a list of neuron IDs[14] deemed to be completely traced by expert proofreaders. Since the ground-truth is comprised of voxel data rather than skeletons, we report only VoI. Further details can be found in Supplementary Section E.

A.2.2.2 FIB-25

The FIB-25 dataset, produced by Takemura et al. (2015), is another FIB-SEM volume imaged at 8nm resolution, containing $\sim 1.8 \times 10^5$µm^3 of raw data taken from the *Drosophila* visual system. Four volumes with ~160µm^3 of labeled data[14] were used for training.

We predicted on the full raw data and then restricted supervoxels to an irregularly shaped neuropil mask[7]. Agglomeration was done in the same fashion as the aforementioned volumes. We created segmentations in two ways: for the first method, following the procedure described in Januszewski et al. (2018), we segmented neurons within the entire neuropil mask. For the second method, we limited segmentation to two sub-RoIs contained within both the neuropil mask and testing region (sections 5074–7950). The sub-RoIs have a size of $\sim 2.2 \times 10^3$µm^3 and $\sim 2.6 \times 10^3$µm^3, respectively. For FFN, we cropped the provided segmentation[7] and relabeled connected components.

Evaluation was limited to a list of proofread, voxel ground-truth labels[14] contained inside the testing region. Depending on the segmentation method, connected components on both ground-truth and segmentation volumes were either cropped and relabelled or untouched. Since the ground-truth is comprised of voxel data rather than skeletons, we report only VoI. Further details can be found in Supplementary Section E.

A.2.3 Neuron Segmentation Accuracy

A.2.3.1 Hemi-Brain

We observe a similar variability of method rankings over RoI sizes on the HEMI-BRAIN dataset.

On the largest investigated RoI (22µm edge length), ACLSD clearly performs best among all affinity-based methods (VoI sum of 0.307 vs. 0.525 for the second best, see Extended Data Figure 2.C). As such, ACLSD is competitive with FFN (VoI sum 0.279), with the notable difference of performing more merge errors, but substantially less split errors than FFN.

On the 17µm RoI, ACLSD again performs better than all other affinity-based methods and also better than FFN (VoI sum of 0.251 vs. 0.371, see Extended Data Figure 2.B). MTLSD is on par with ACLSD on this RoI, which stands in contrast to its substantially worse performance on the 22µm RoI. This observation further puts into question to what extent the accuracy of a segmentation can be extrapolated from smaller to larger RoI sizes.

These concerns become even more evident when turning to the results on the smallest RoI of 12µm edge length. Here, ACLSD performs worse than all other methods, with a substantial margin to the best performing method, MTLSD (VoI sum 0.197 vs 0.085). Even BASELINE achieves very good results on this RoI (VoI sum 0.102), although it would be a poor choice in production given its detrimental performance on the larger RoIs. We have to conclude that the size of this RoI is likely not large enough to accurately deduce whether the differences in method performance are due to model accuracies or data biases.

A somewhat surprising result is the performance of AcRLsd on this dataset. Although architecturally very similar to AcLsd (the only difference is that AcRLsd receives raw data and LSDs in the second pass, while AcLsd receives only LSDs), AcRLsd is substantially worse on the two larger RoIs. This stands in contrast to the results we obtained on the Zebrafinch dataset. Our results do not allow us to say with confidence whether this artifact is due to overfitting to the training data (which might be more likely to happen for AcRLsd) or due to model noise introduced by the random initialization during training.

A.2.3.2 FIB-25

We first evaluated all methods on the full testing RoI of the Fib-25 dataset (Extended Data Figure 2.D). On the full RoI, LSDs generally do not perform well. We observe that the best auto-context method (AcLsd) performs worse than the Baseline (VoI sum 1.413 vs 1.355). MtLsd achieves higher accuracy than both auto-context networks. FFN exceeds all other methods (VoI sum 1.056)[4], and Malis is not far behind (VoI sum 1.061). Since those results are not consistent with the results seen on the Zebrafinch and Hemi-brain volumes, we visually inspected the segmentations. We found a high rate of false merges occurring in the periphery of the testing RoI, stemming from nuclei and boundaries of the imaged volume, which are not contained in the training data. As such, the full testing RoI of this dataset favors "conservative" methods, *i.e.*, methods that have higher split rates.

To test the plain neuropil segmentation accuracy, we further cropped two RoIs (~$2.2 \times 10^3 \mu m^3$ and ~$2.6 \times 10^3 \mu m^3$) inside the testing region, such that they contain only dense neuropil (see Supplementary Section E.3.3 for details).

On the two sub RoIs, LSDs outperform other affinity-based methods and are comparable to FFN, (Extended Data Figure 2.E,F). Consistent with the Zebrafinch and Hemi-brain results, using an auto-context approach seems to generate the best results. On sub RoI 1, AcLsd slightly exceeds the accuracy of FFN (VoI sum: 0.625 vs 0.700, respectively). We observe similar results on sub RoI 2 (VoI sum: 0.761 vs 0.780, respectively).

These results highlight the need for masking neuropil when processing large volumes, as was done in the Zebrafinch dataset. LR affinities perform poorly across RoIs, which might suggest that an increased affinity neighborhood is sometimes not sufficient for improving direct neighbor affinities.

A.3 Discussion

A.3.1 Metric Evaluation

An important but challenging task is finding a robust metric for assessing the quality of a neuron segmentation. Ideally, such a metric reflects the amount of time needed to proofread a segmentation. Here, we presented results in terms of VoI and ERL, two commonly used metrics for this task.

VoI directly reports the amount of split and merge errors. Being a voxel-wise metric, however, VoI can be sensitive to slight, but systematic, shifts in boundaries. At the same time, small topological changes might go unnoticed, which is especially problematic in fine neurites in the vicinity of synapses (Funke et al., 2017).

ERL reports the expected error-free path-length of a reconstruction with respect to skeleton ground-truth. Similar to VoI, ERL is not sensitive to small topological changes close to terminals. Furthermore, ERL disproportionately punishes merge errors and subsequently favors split-preferring methods, explained in Results (Plaza and Funke, 2018). Additionally, we found that ERL increases non-monotonically with varying volume sizes (Figure 4.B), which is due to the fragmentation of skeletons in volumes that are not large enough to contain entire neurons.

Consequently, neither method directly reflects the labor required for proofreading a segmentation, which is arguably the relevant quantity to optimize (Plaza, 2016; Funke et al., 2017). This quantity depends on the available tools for proofreading, and in particular on the amount of interactions needed to fix errors of different kinds: False splits might be hard to find, but do require only one interaction to merge. False merges, on the other hand, might be easy to spot, but the number of interactions needed to fix them depends greatly on the proofreading tool. Current proofreading tools (Zhao et al., 2018; Dorkenwald et al., 2022) allow annotators to correct merge errors with a few interactions. We therefore introduced the MCM, a metric which uses graph cuts to emulate the amount of interactions required to correct false merges in a segmentation. We observed a linear growth of MCM with volume size (Figure 4.D), which is a necessary condition for neuron segmentation metrics that measure the amount of proofreading effort needed (assuming an equal distribution of errors).

Unfortunately, MCM is computationally quite expensive. The sequence of graph-cuts needed for the evaluation of merge errors quickly becomes infeasible on large volumes. However, MCM shows general ranking agreement with VoI, evaluated on 11 randomly sampled sub-RoIs in Zebrafinch (Supplementary Figure 4) and across different thresholds (Supplementary Figure 7). These findings suggest that VoI can serve as a reasonable proxy to rank methods based on their expected proofreading time.

Additionally, we find VoI to be a robust metric for the validation of method parameters: For each affinity-based network, the threshold minimizing VoI sum on the validation set is also close to the best threshold on the testing set (Supplementary Figure 5). This property is of practical relevance, as in any real-world scenario hyperparameters have to be adjusted on a volume that is substantially smaller than the target volume. Unfortunately, ERL does not seem to exhibit this property to the same degree: the best validation thresholds gradually diverge from the best testing thresholds as scale increases (Supplementary Figure 6), which makes it difficult to extrapolate segmentation accuracy from a validation dataset.

A.3.2 Auxiliary Learning for Boundary Prediction

Surprisingly, we see that LR affinities do not perform as well across the investigated datasets. While LR affinities share some of the benefits of LSDs, they might not be as efficient as LSDs in encoding higher-level features. For example, LR affinities have blind spots (missing neighborhood steps), whereas LSDs are spatially homogeneous. Additionally, we found LR affinities to be detrimental when used with masking of glia and other structures. It is likely harder to correlate nearest neighbor affinities with a long-range neighborhood in the presence of masks.

While Lee et al. (2017) saw superhuman accuracy using an increased affinity neighborhood on the SNEMI3D challenge, the processed volume was relatively small (~$110 \mu m^3$). Our results suggest that it is hard to correlate accuracy on small volumes to

[4]The authors of Januszewski et al. (2018) report a VoI split of 0.8837. While we were able to replicate the reported VoI merge score of 0.053, we found VoI split to be 1.003.

accuracy on large volumes (Figure 1.A)[5]. Additionally, we only consider an increased affinity neighborhood and not other aspects of the original LR implementation, such as residual modules in the U-NET and inference blending, which might be essential for further performance increases. Finally, the SNEMI3D dataset has an anisotropy factor of ~5, whereas the data we test on here has an anisotropy factor of either ~2 (ZEBRAFINCH) or is isotropic (HEMIBRAIN, FIB-25).

A.3.3 Auto-Context Refinement

It remains unclear whether auto-context is always necessary. While it does consistently yield optimal results on large datasets, it is likely unnecessary for small to intermediate sized volumes, due to the increase in computation. Since the MTLSD network still offers improved accuracy over BASELINE affinities on every investigated dataset, it is probably sufficient in the majority of cases. In this context, a multi-task network can be considered the default configuration while an auto-context approach should be considered in complex cases where the former would otherwise fail. As always, the optimal solution would ideally be chosen based on an available validation set, as was done for the ZEBRAFINCH.

A.3.4 Masking

In addition to using masks during post-processing, masking of irrelevant structures can be incorporated in the training process. The ZEBRAFINCH training volumes already had some glial processes masked out. We trained all networks to predict zero affinities in these regions (see Supplementary Figure 12 for a visualization). We then discarded fragments with close to zero affinity values during agglomeration. Methods which succeeded in learning to mask these areas (BASELINE, MTLSD, ACLSD, ACRLSD) produced better results than those that did not (MALIS, LR).

A.4 Conclusions and Future Directions

Although adding LSDs as an auxiliary learning task substantially increases accuracy, it is unclear whether different shape descriptors could lead to further improvements. The LSDs proposed here were subjectively engineered based on features that we expected to be important to encode object shape. Future experiments could incorporate different features or focus on learning an optimal embedding rather than a hand-designed one. In that context, we note that it is not clear whether each component of the LSD embedding contributes equally to the improvement of affinity predictions.

Currently, we only use LSDs as an auxiliary learning task. As a result, affinities are still required to produce a segmentation. Whether this is really needed is an open question, since the predicted LSDs already identify objects reliably. An interesting future direction would be to use the predicted local shape information directly for fragment agglomeration. As an intermediate step, LSDs can serve to provide a second source of information for identifying errors in a segmentation. Once a segmentation is generated, LSDs could be calculated on the labels and then compared with the initial LSD predictions. The difference between the two would likely highlight regions containing errors (Supplementary Figure 16.C).

LSDs were designed for the goal of neuron segmentation but might also be applicable to other instance segmentation problems. As an example, we found that the LSDs improve segmentations on plant epithelial cells (Supplementary Figure 16.E, Supplementary Table 8) and perform well on cell bodies and mitochondria (Supplementary Figure 16.A,B). Since the LSDs are computed inside a Gaussian constrained to each object, the vectors allow for smooth transitions on both spherical and elongated shapes. In general, we believe that objects that have a blob-like structure such as other organelles and various cell types would likely benefit from LSDs. Furthermore, the direction vectors of the LSDs provide insight into neuropil vs. tract regions of the brain (Supplementary Figure 16.D). These predictions could be leveraged in order to generate better tissue masks. While the LSDs presented here were conceived for a specific instance segmentation task, it would be interesting to see the LSDs extended and applied to other microscopy problems.

[5]Also shown by the authors of Januszewski et al. (2018) in supplementary tables 4 and 5.

B LOCAL SHAPE DESCRIPTORS

We define the notational shorthand

$$\mathrm{f}^i_k(v) = v_k\, \mathrm{b}^i(v) \qquad k \in \{x,y,z\} \tag{1}$$
$$\mathrm{f}^i_{kl}(v) = v_k v_l\, \mathrm{b}^i(v) \qquad k,l \in \{x,y,z\}, \tag{2}$$

and use those to rewrite

$$\begin{aligned} \mathrm{m}^i_k(v) &= \frac{\left(v_k\, \mathrm{b}^i * \mathrm{w}\right)(v)}{\left(\mathrm{b}^i * \mathrm{w}\right)(v)} && k \in \{x,y,z\} \\ \mathrm{c}^i_{kl}(v) &= \frac{\left(v_k v_l\, \mathrm{b}^i * \mathrm{w}\right)(v)}{\left(\mathrm{b}^i * \mathrm{w}\right)(v)} - \mathrm{m}^i_k(v)\, \mathrm{m}^i_l(v) && k,l \in \{x,y,z\} \end{aligned} \tag{3}$$

as follows:

$$\mathrm{m}^i_k(v) = (\mathrm{f}^i_k * \mathrm{w})(v)/\mathrm{s}^i(v) \qquad k \in \{x,y,z\} \tag{4}$$
$$\mathrm{c}^i_{kl}(v) = (\mathrm{f}^i_{kl} * \mathrm{w})(v)/\mathrm{s}^i(v) - \mathrm{m}^i_k(v)\, \mathrm{m}^i_l(v) \qquad k,l \in \{x,y,z\}. \tag{5}$$

It can be seen that (4) is equal to the local center of mass, limited both by the object mask b and the local window w:

$$\begin{aligned} \mathrm{m}^i_k(v) &= (\mathrm{f}^i_k * \mathrm{w})(v)/\mathrm{s}^i(v) \\ &= \frac{1}{\mathrm{s}^i(v)} \sum_{v' \in \Omega} \mathrm{f}^i_k(v')\, \mathrm{w}(v - v') \\ &= \frac{1}{\mathrm{s}^i(v)} \sum_{v' \in \Omega} v'_k\, \mathrm{b}^i(v')\, \mathrm{w}(v - v') \end{aligned}$$

Similarly, the computation of the local covariance of voxel coordinates is equivalent to a convolution of the local window w with $\mathrm{f}^i_{kl}(v)$. The local covariance is defined as:

$$\begin{aligned} \mathrm{c}^i_{kl}(v) &= \frac{1}{\mathrm{s}^i(v)} \sum_{v' \in \Omega} (v'_k - \bar{v}_k)(v'_l - \bar{v}_l)\, \mathrm{b}^i(v')\, \mathrm{w}(v - v') \\ &= \frac{1}{\mathrm{s}^i(v)} \sum_{v' \in \Omega} (v'_k - \mathrm{m}^i_k(v))(v'_l - \mathrm{m}^i_l(v))\, \mathrm{b}^i(v')\, \mathrm{w}(v - v') \\ &= \frac{1}{\mathrm{s}^i(v)} \sum_{v' \in \Omega} \left(v'_k v'_l - v'_k\, \mathrm{m}^i_k(v) - v'_l\, \mathrm{m}^i_k(v) + \mathrm{m}^i_k(v)\, \mathrm{m}^i_l(v)\right) \cdot \\ &\qquad \cdot \mathrm{b}^i(v')\, \mathrm{w}(v - v') \end{aligned}$$

Rearranging terms reveals that $c^i_{kl}(v)$ can efficiently be computed via a convolution as well:

$$c^i_{kl}(v) = \frac{1}{s^i(v)}\Bigg(\sum_{v' \in v} v'_k v'_l \, b^i(v') \, w(v - v') - m^i_l(v) \sum_{v' \in v} v'_k \, b^i(v') \, w(v - v') - m^i_k(v) \sum_{v' \in v} v'_l \, b^i(v') \, w(v - v') + m^i_k(v) \, m^i_l(v) \sum_{v' \in v} b^i(v') \, w(v - v') \Bigg)$$

$$\begin{aligned} c^i_{kl}(v) =& \frac{1}{s^i(v)}\Bigg(\sum_{v' \in v} \underbrace{v'_k v'_l \, b^i(v')}_{f^i_{kl}(v')} w(v - v') - \\ & m^i_l(v) \underbrace{(f^i_k * w)(v)}_{m^i_k(v)\, s^i(v)} - \\ & m^i_k(v) \underbrace{(f^i_l * w)(v)}_{m^i_l(v)\, s^i(v)} + \\ & m^i_k(v)\, m^i_l(v)\, s^i(v) \Bigg) \\ =& \frac{1}{s^i(v)} \Big((f^i_{kl} * w)(v) - s^i(v)\, m^i_l(v)\, m^i_k(v) \Big) \\ =& (f^i_{kl} * w)(v) / s^i(v) - m^i_l(v)\, m^i_k(v) \end{aligned}$$

C Min-Cut Metric

The Min-Cut Metric (MCM) measures the number of edit operations that need to be performed by a human annotator in a hypothetical proofreading tool that allows to: (1) *merge* wrongly split segments and (2) *split* wrongly merged segments by means of a min-cut[6]. To this end, we assume that the segmentation to interact with results from an agglomeration of fragments (or "supervoxels"). In particular, we assume that a *fragment graph* $G = (V, E, s)$ is available, where each node $v \in V$ corresponds to a fragment and edges $(u, v) \in E$ are introduced between neighboring fragments. Each edge $e \in E$ has an associated *merge score* $s(e)$, which denotes under which agglomeration threshold the two incident fragments are to be merged into the same segment. A segmentation of the fragment graph is induced by a merge score threshold θ. Let $E_\theta = \{e \in E \mid s(e) \leq \theta\}$ be the set of *filtered edges*. Each connected component in the graph $G_\theta = (V, E_\theta)$ then corresponds to one segment. We will refer to the segment ID of a fragment under a given threshold θ as $l_\theta(v)$.

For the MCM metric, we assume ground-truth is available in the form of *skeletons*. Let T be the set of ground-truth skeletons, with each $t \in T$ being a set of skeleton nodes. We will refer to the skeleton ID of a skeleton node a as $s(a)$ and the fragment underlying the skeleton node as $l_\theta(a)$.

Given a segmentation l_θ, MCM first simulates the splitting of all wrongly merged structures as given by ground-truth skeletons. For that, we first identify *merging* segments, *i.e.*, segments that contain nodes from more than one skeleton. For each merging segment, we iteratively perform a series of min-cuts through the fragment graph, until the skeletons are separated. For that, we repeatedly find a pair of skeleton nodes a and b, such that

1) $s(a) \neq s(b)$ (the skeleton nodes belong to different skeletons),
2) $l_\theta(a) = l_\theta(b)$ (the underlying fragments belong to the same segment), and
3) the Euclidean distance between a and b is minimized.

We then perform a min-cut on the fragment graph, with u and v as the source and sink, respectively, and the capacity $c(e)$ of edges e in the fragment graph set proportional to $-s(e)$, such that edges with a high merge score are cheaper to cut. Once the min-cut is found, all edges of the cut are removed from E_θ and the segmentation is updated accordingly. For a visualization of this procedure, see Extended Data Figure 1.A. This procedure aims to mimic a proofreader, who identified a merge and consequently picked two close locations on either side of the merge to perform a split operation.

In some cases, a min-cut can fail to separate all nodes of the merged skeletons with a single cut from two selected nodes. In this case, the procedure is repeated until all skeletons are separated, leading to additional split errors (see Extended Data Figure 1.B for an example).

After all skeletons are separated, the remaining split errors are counted. For that, we assume that each split requires one merge operation to be fixed. More generally, we identify the segments underlying each skeleton t: Let $L(t) = \{l_\theta(a) \mid a \in t\}$ be the set of segments underlying a skeleton. The number of required merge operations is then recorded as $1 - |L(t)|$.

[6]Also referred to as "cleaving".

D Zebrafinch

D.1 Training

D.1.1 Data

33 volumes of densely labeled neurons[7] were used for training. Each volume was padded with raw data. 30 volumes had raw dimensions of ~7, 4.95, 4.95µm (zyx) and label dimensions of ~3, 1.35, 1.35µm. The remaining 3 volumes had raw dimensions of ~6.6, 5.9, 5.9µm and label dimensions of ~2.6, 2.3, 2.3µm. Some regions containing glial processes were already set to zero and incorporated during network training (Supplementary Figure 11.A). A labels mask (1 inside labels RoI, null outside) was generated and used during training.

D.1.2 Networks

All methods used the U-Net architecture described in Funke et al. (2019). Networks consisted of three layers and were downsampled by a factor of [1,3,3] in the first two layers and [3,3,3] in the last layer. The reverse was done for the upsampling path. 12 initial feature maps were used and features were multiplied by a factor of 5 between layers. The resulting data was further convolved and passed through a sigmoid activation to get from 12 output feature maps[8] to either 3 (affinities) or 10 feature maps (LSDs). All networks used an MSE loss, minimized with an Adam optimizer. The Malis network was trained to 10k iterations using MSE to initialize affinities and was then switched to Malis loss for the remainder of training.

Non auto-context networks had an input shape (raw) of [84,268,268] and output shape (labels, LSDs, affinities) of [48,56,56] (voxels, zyx). Auto-context networks had an input

[7]Kindly provided by the authors of Januszewski et al. (2018)

[8]MtLsd network had 14 output feature maps to account for 13 final feature maps from the affinities (3) and LSDs (10)

shape (raw) of [120,484,484], an intermediate shape (predicted LSDs) of [84,268,268], and an output shape (labels, affinities) of [48,56,56] (see Supplementary Figure 13.A for visualization of auto-context training shapes on Fib-25). The predicted LSDs used in the intermediate shape were taken from a pre-trained network which predicted LSDs from raw. Non auto-context networks were trained to 400k iterations. Auto-context networks were trained to ~200k iterations following the 400k iterations of LSD training. See Supplementary Table 9 for a breakdown of the MtLsd network as an example.

All networks used a single voxel affinity neighborhood [1,1,1]. The LR network used three additional neighborhood steps of [3,3,3], [5,9,9] and [15,27,27]. The computed LSDs used a sigma of 120 nm and a downsampling factor of two.

D.1.3 Pipeline

Each training batch was randomly picked from one of the 33 training volumes. For each batch, the raw data was first normalized and padded with zeros. Labels were padded with the maximum padding required to contain at least 50% of ground-truth data assuming a worst case rotation of 45°. Data was randomly sampled from each dataset using a labels mask to ensure every batch contained at least 50% of ground-truth data. Data was then augmented with elastic transformations, random mirrors + transposes, and intensities (see Supplementary Table 9 for augmentation hyper-parameters used for example MtLsd network). The following was done to the respective networks:

- **Baseline**, **LR** - Label boundaries were first eroded by a single voxel. Ground truth affinities were calculated on the labels using the pre-defined affinity neighborhoods and a scale array was created to balance loss between class labels. Training: [raw + gt affs] → pred affs.
- **Malis** - Label boundaries were eroded. If training loss was in Malis phase (*i.e.* after 10k iterations), connected components were relabelled before calculating ground-truth affinities. If training loss was in MSE phase (*i.e.* before 10k iterations), labels were subsequently balanced. Training: [raw + gt affs] → pred affs.
- **LSDs** - Ground truth LSDs were calculated on the labels using the pre-defined sigma and downsampling factor. Training: [raw + gt LSDs] → pred LSDs.
- **MtLsd** - Label boundaries were eroded. Ground truth LSDs were calculated followed by ground-truth affinities. Labels were then balanced. Training: [raw + gt LSDs + gt affs] → [pred LSDs + pred affs].
- **AcLsd**, **AcRLsd** - Label boundaries were eroded, ground-truth affinities were calculated, and labels were balanced. LSDs were then predicted in a slightly larger region and used as input to train the affinities. Training: [raw + gt LSDs] → pred LSDs → pred affs. For AcRLsd, cropped raw was incorporated in the second pass, in addition to predicted LSDs.

This process was repeated for a pre-defined number of iterations (generally until loss convergence).

D.2 Prediction

Prediction was done in a block-wise fashion restricted to the Benchmark Roi. Individual workers used Gunpowder[9] to predict output data (*i.e.* affinities or LSDs) and were distributed throughout the volume with DaisyNguyen et al. (2022). Block size was chosen with respect to how much data could fit in GPU memory. Most networks had a smaller block size in order to fit on 2080 RTX GPUs (~12 GB RAM). While this increased the total number of blocks to process, the amount of workers available to use was sufficient to minimize total processing time. The auto-context networks were too large to fit on 2080 RTX GPUs and were therefore run on Tesla V100 GPUs. While there were less V100's available, the block size could be greatly increased (~32 GB RAM), decreasing the total amount of blocks to process. LSDs were physically written to file before use in the auto-context networks. This was done for visualization; prediction could be adapted to generate LSDs on the fly. All networks wrote affinities to file and then subsequently used them for segmentation.

D.3 Segmentation

D.3.1 Watershed

Seeded watershed[10] was done on the affinities generated during prediction. Both non-masked and neuropil-masked supervoxels were produced. Due to data anisotropy, supervoxels were extracted for each section separately. An epsilon agglomeration was used to agglomerate fragments to a predefined threshold (0.1). This was done to decrease the number of RAG nodes during the full agglomeration step. Supervoxels which had an average affinity value lower than a pre-defined threshold (0.05) were filtered out of the RAG and set to zero in the resulting datasets. A block size of 3.6µm^3 and context of [12,27,27] voxels (zyx) were used.

D.3.2 Agglomeration

Supervoxels were agglomerated using hierarchical region agglomeration[10] in which edges with lower affinity scores are merged earlier. We empirically chose to use both 50 and 75 quantile merge functions since they produced the best results in Funke et al. (2019). The same block size and context as watershed were used.

D.3.3 Segment

The center point of the Benchmark Roi was used to grow sub RoIs. The first sub RoI was created by growing the center point in each dimension (positive and negative) by the block size used during watershed and agglomeration. This resulted in 10.8µm edge lengths (3.6µm + (3.6 x 2)). This RoI was again grown by the block size to produce an RoI with 18µm edge lengths (10.8µm + (3.6 x 2)). This was repeated for a total of 10 RoIs (in addition to the Benchmark Roi). Segmentations were created for each RoI by cropping the RAG and relabelling connected components on the graph[11]. This was done over a range of thresholds for each network (threshold range = [0 - 1], step size = 0.02, total thresholds = 50). Segmentations were created for both masked and non-masked data and both merge functions.

D.4 Evaluation

Manually traced skeletons[7] (12 validation, 50 testing) were used for evaluation. For each sub RoI, skeletons were cropped, either masked to neuropil or not masked, and connected components were relabelled[11]. For affinity-based methods, fragment ids were first mapped to skeleton ids in each block. This mapping was then used

[9] http://funkey.science/gunpowder

[10] https://github.com/funkey/waterz

[11] https://github.com/funkelab/funlib.segment

to assign segment ids to skeleton ids for each threshold, using the lookup tables generated in Supplementary Section D.3.3. A site mask was used to restrict segments to the skeleton nodes. The resulting node - segment mapping was used to compute[12] ERL, NERL and VoI.

Additionally, on the first three sub RoIs, the MCM was calculated using masked skeletons and segmentations. For the FFN, a single segmentation[7] was used to generate the node - segment mappings. The full segmentation was downloaded[13] and cropped to each sub RoI, either masked or not masked, and connected components were relabelled. Only ERL, NERL and VoI were calculated as there were no supervoxels to use for the MCM. For all affinity-based methods, we repeated these steps using the validation skeletons on the benchmark RoI. The optimal thresholds indicated in test set plots were determined by the thresholds which minimized VoI (for VoI and MCM plots) and maximized ERL (for ERL plots).

E FIB-SEM VOLUMES

E.1 Training

E.1.1 Data

HEMI-BRAIN: 8 volumes of densely labeled neurons[14] were used for training. Volumes were taken from various neuropils[15] contained within the dataset generated in (Scheffer et al., 2020). The LOBULA PLATE and LATERAL HORN volumes contained ~4µm^3 of raw data and ~2µm^3 of labeled data, while the others contained ~6µm^3 of raw data and ~4µm^3 of labeled data (Supplementary Figure 11.B).

FIB-25: 4 volumes of densely labeled neurons[14] were used for training. The labels were not padded with raw as was done in the ZEBRAFINCH and HEMI-BRAIN volumes. Two volumes contained ~4µm^3 of raw / labeled data, and two volumes contained 2µm^3 of raw / labeled data (Supplementary Figure 11.C). Label masks were generated for all volumes, as done in the ZEBRAFINCH.

E.1.2 Networks

Networks consisted of same architecture as ZEBRAFINCH networks except downsampling was isotropic with a factor of [2,2,2] in the first two layers and [3,3,3] in the last layer. Features were multiplied by a factor of 6 between layers.

Non auto-context networks had an input shape (raw) of [196,196,196] and output shape (labels, LSDs, affinities) of [92,92,92]. Auto-context networks had an input shape (raw) of [304,304,304], an intermediate shape (predicted LSDs) of [196,196,196], and an output shape (labels, affinities) of [92,92,92]. Non auto-context networks were trained to 400k iterations. Auto-context networks were trained to ~300k iterations following 400k iterations of LSD training. See Supplementary Table 10 for a breakdown of the MTLSD network as an example.

All networks used a single voxel affinity neighborhood [1,1,1]. The LR network used three additional neighborhood steps of [3,3,3], [5,5,5] and [13,13,13]. The computed LSDs used a sigma of 80 nm and a downsampling factor of two.

[12]https://github.com/funkelab/funlib.evaluate

[13]https://github.com/seung-lab/cloud-volume

[14]Kindly provided by the Janelia FlyEM project team (https://janelia.org/project-team/flyem)

[15]ELLIPSOID BODY: 2, PROTOCEREBRAL BRIDGE: 2, FAN-SHAPED BODY: 2, LOBULA PLATE: 1, LATERAL HORN: 1

E.1.3 Pipeline

All networks were trained following the same pipeline as the ZEBRAFINCH networks using either 8 (HEMI-BRAIN) or 4 (FIB-25) ground-truth volumes. The augmentations were computed isotropically, in contrast to the ZEBRAFINCH networks (see Supplementary Table 10 for augmentation hyper-parameters used for example MTLSD network). For FIB-25 training, affinities and LSDs were masked at the boundaries to ensure that prediction on the irregularly shaped FIB-25 volume did not include boundary artifacts (Supplementary Figure 13.B).

E.2 Prediction

For the HEMI-BRAIN, prediction was restricted to the three RoIs described in Section A.2.2.1. For FIB-25, prediction was done on the full FIB-25 volume (including the background). The process was the same as in the ZEBRAFINCH.

E.3 Segmentation

E.3.1 Watershed

Fragment extraction was performed isotropically and used no epsilon agglomeration step or mean affinity filtering, in contrast to the ZEBRAFINCH. A block size of 3µm^3 and context of 31 voxels were used. For the HEMI-BRAIN, watershed was done on each predicted RoI and restricted using an ELLIPSOID BODY mask[14]. For FIB-25, an irregularly shaped tissue mask[7] was used[16].

E.3.2 Agglomeration

Agglomeration was done using the same merge functions on the ZEBRAFINCH. The same block size and context from watershed were used.

E.3.3 Segment

For the HEMI-BRAIN, segmentations were created for the three processed RoIs. For FIB-25, segmentations were created for the full FIB-25 RoI and two sub RoIs. The same threshold range from the ZEBRAFINCH was used.

E.4 Evaluation

HEMI-BRAIN: dense ground-truth[14] and an FFN segmentation[7] were available for the entire HEMI-BRAIN. Both volumes were downloaded[17,18] and cropped to the three established RoIs. The cropped datasets were then constrained to the ELLIPSOID BODY. The ground-truth was filtered using a whitelist of proofread ids. Connected components were relabelled and boundaries were slightly eroded. **FIB-25**: dense ground-truth[14] and an FFN segmentation[7] were already cropped to the testing RoI. The ground-truth was already filtered with a whitelist and boundaries were already eroded. Both volumes were further cropped to the two sub RoIs and connected components were relabelled. For affinity-based methods, VoI was calculated between the consolidated ground-truth and segmentations over all thresholds on each RoI. For the FFN, VoI was calculated on the single segmentation for each RoI.

F Throughput

For each affinity-based network, we calculated the amount of floating point operations (FLOPs) for the processing of one block[19] using TensorFlow's Profiler[20] (see Supplementary Table 11 for a breakdown by operation). From the computed FLOPs and the block size, we derived FLOPs/µm. For FFN, FLOPs are reported for the full Zebrafinch RoI in Januszewski et al. (2018), which we divided by the full RoI volume to get FLOPs/µm.

We chose to use FLOPs rather than runtime to evaluate the computational cost of each method. The runtime for affinity based-methods is likely to be a noisy measurement because it does not consider data input/output (I/O). For each block, data loading is first done on the CPU before being transferred to the GPU, making the throughput dependent on factors such as chunk size and compression. Runtime is also likely dependent on factors relating to a given compute cluster (such as the file system and networking). A lot more effort could be spent on optimizing the runtime performance, and it is not clear how much effort was spent on optimizing FFN in this regard. FLOPs therefore provides a more reliable readout of computational performance when considering the operations required for each method. For the processing of future petabyte-sized datasets, it will be imperative to optimize for both network architecture and data I/O.

G Extended Experiments

G.1 Serial Section Data (ssTEM)

To test the efficacy of LSDs on both ssTEM and mouse neural tissue, we used the publicly available data from (Microns Consortium et al., 2021). To train the networks, we used 38 volumes (~908µm^3 total, ~24µm^3 average) taken from three datasets (Basil, Minnie, Pinky). Several volumes were excluded from training and testing due to overlap. The volumes were either imaged at 40x4x4 or 40x8x8 nanometer resolution (zyx). We therefore chose to downsample volumes by a factor of 2 laterally to ensure consistency. Networks were trained similarly to the Zebrafinch networks with added missing and shifted section augmentations.

Due to the anisotropy of the data and valid padding of the networks, we opted to use 2D convolutions in the lowest level of the U-Net. We trained Baseline, LR, MtLsd, AcLsd, and AcRLsd networks and omitted Malis following the results on the block face datasets. A foreground mask was provided (zero in missing or broken sections) and used to ensure that predictions persisted through these regions.

Evaluation was done on 4 test volumes (~232µm^3 total, ~58µm^3 average). Watershed and agglomeration was done similarly to the Zebrafinch (with the exception of a neuropil mask). Ground truth neurons were relabelled between missing sections to gauge neuropil performance since we did not engineer ssTEM-specific preprocessing (improved alignment, duplicated section augmentations, etc.)

Our results suggest that the LSDs also work well on serial section data and would likely benefit from the same processing as other methods. They look qualitatively reasonable (Supplementary Figure 1) and when considering pure neuropil, they seem to outperform baseline methods (Supplementary Table 6). Consistent with the other datasets, using an autocontext network produces the highest accuracy (AcLsd VoI of 0.639). Due to the small size of these datasets, it is difficult to extrapolate accuracy to larger volumes but we expect that the LSDs would generally help to improve Baseline affinities.

G.2 Ablations

In order to see how important each component of the LSDs is, we ran an ablation experiment in which we limited the embedding to each possible combination of components for both an MtLsd network and AcLsd network. Networks were trained on the Hemi-brain training data and tested on the 12 µm RoI.

We expected that the MtLsd networks would not be substantially affected by removing components since they still learn the affinities in the same pass. We found that to generally be true, with the results not indicative of a single superior and inferior combination (Supplementary Table 7). Conversely, we hypothesized that the AcLsd networks would be highly dependent on which components of the LSDs were fed into the second pass. We found that, generally speaking, any combination of LSDs will generate reasonable results but using only the size component as input to the second pass produces poor results (Supplementary Table 7). This is illustrated in Supplementary Figure 2, where it is clear that the affinities and subsequent segmentations do benefit from a combination of components that is not limited to the size of the neural process. It is also not clear how much the numbers are due to random training noise.

G.3 Non-EM Data: Epithelial Cells

We tested the LSDs on the publicly available plant epithelial ovule dataset from Wolny et al. (2020). We used 20 volumes for training (~6706µm^3 total, ~335µm^3 average). All volumes were cropped in the z dimension to sections that contained labels (i.e, volume ends that were completely background were removed). We implemented networks as done for the ssTEM extended experiment, but found results to be poor when using hierarchical agglomeration due to the challenging nature of the data. To overcome this, we decided to use the mutex watershed (Wolf et al., 2018). To this end, we did not consider Baseline affinities since a long range affinity neighborhood is required for the mutex watershed. Additionally, we found MtLsd results using a long range affinity neighborhood to be qualitatively poor, likely due to the network trying to learn too much simultaneously.

We therefore limited our evaluation to LR affinities as a baseline, and an AcLsd network. In contrast to the Zebrafinch, Fib-25, and Hemi-brain experiments, our long range neighborhood also consisted of diagonal affinities as they are useful for the mutex watershed. We used 7 volumes for testing (~2466µm^3 total, ~352µm^3 average). Following the mutex watershed, we filtered out small objects (500 voxels minimum size) and expanded the labels to fill the intermediate space between objects. Since some of the ground truth was not fully labeled, we restricted our segmentations to a foreground mask generated from the ground truth. Finally, we relabelled connected components. Results suggest that the LSDs are useful for non-EM data, and seem to work especially well when combined with the mutex watershed (Supplementary Table 8), a promising indication for future work. The results are also qualitatively appealing (Supplementary Figure 16.E).

[16]Mask contains some background and cell bodies

[17]https://github.com/janelia-flyem/dvid

[18]https://github.com/janelia-flyem/neuclease

[19]One block is defined as the largest output volume that can be predicted by a network in one pass on the respective GPU it was evaluated on.

[20]https://www.tensorflow.org/guide/profiler

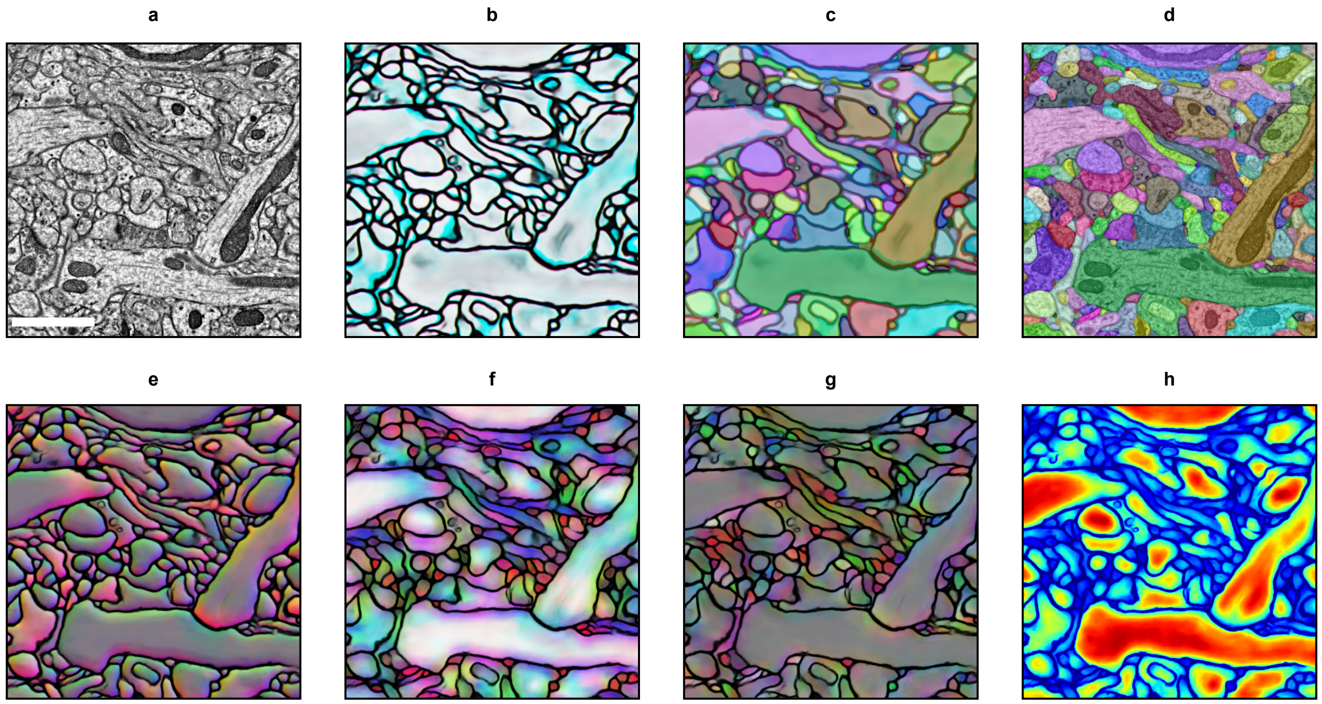

Supplementary Figure 1: LSD results on example ssTEM data. **A**. Raw EM data, scale bar = 1 µm. **B**. Affinities computed from second pass of AcLsd network. **C**. Segmentation produced from affinities. **D**. Segmentation overlayed with raw data. **E,F,G,H**. Mean offset, diagonal entries of covariance, off-diagonal entries of covariance, and size components of LSDs.

Dataset	Imaging Method	Tissue	Resolution (xyz)	Training Data	Testing Data
Zebrafinch	SBFSEM	songbird	9x9x20 nm	33 dense volumes (~ 200 μm^3)	50 skeletons (97 mm)
Hemi-brain	FIBSEM	*Drosophila*	8x8x8 nm	8 dense volumes (~ 450 μm^3)	3 whitelisted volumes (~ 1.7×10^4 μm^3)
Fib-25	FIBSEM	*Drosophila*	8x8x8 nm	4 dense volumes (~ 160 μm^3)	1 whitelisted volume (~ 7.7×10^3 μm^3)

Supplementary Table 1: Overview of datasets used in study

(A) Validation = Best VoI Sum

Method	VoI Split	VoI Merge	VoI Sum	ERL (µm)	NERL
Baseline	1.115	2.741	3.856	9.147	0.038
LR	2.072	2.286	4.358	9.517	0.040
FFN	1.068	**1.188**	**2.256**	**16.747**	**0.070**
MtLsd	**0.625**	2.794	3.420	8.855	0.037
AcLsd	1.192	1.222	2.414	12.886	0.054
AcRLsd	0.944	1.346	2.290	12.667	0.053

(B) Validation = Best ERL

Method	VoI Split	VoI Merge	VoI Sum	ERL (µm)	NERL
Baseline	0.757	4.586	5.343	9.113	0.038
LR	0.905	4.851	5.756	9.044	0.038
FFN	1.068	**1.188**	2.256	**16.747**	**0.070**
MtLsd	0.670	2.578	3.247	11.352	0.047
AcLsd	**0.533**	2.021	2.554	11.419	0.047
AcRLsd	0.803	1.436	**2.239**	13.470	0.056

Supplementary Table 2: Best results on Zebrafinch Benchmark Roi

Method	VoI Split	VoI Merge	VoI Sum
Baseline	0.062	0.040	0.102
LR	0.070	0.061	0.131
Malis	0.144	0.036	0.180
FFN	0.129	0.046	0.175
MtLsd	**0.048**	0.037	**0.085**
AcLsd	0.191	**0.006**	0.197
AcRLsd	0.078	0.009	0.087

(A) 12 µm Region of Interest

Method	VoI Split	VoI Merge	VoI Sum
Baseline	0.394	0.725	1.118
LR	0.284	1.040	1.324
Malis	0.523	0.065	0.588
FFN	0.347	**0.024**	0.371
MtLsd	0.211	0.056	0.267
AcLsd	**0.161**	0.090	**0.251**
AcRLsd	0.165	0.404	0.568

(B) 17 µm Region of Interest

Method	VoI Split	VoI Merge	VoI Sum
Baseline	0.345	1.466	1.811
LR	0.143	0.729	0.873
Malis	0.437	0.162	0.599
FFN	0.242	**0.036**	**0.279**
MtLsd	0.166	0.470	0.636
AcLsd	**0.126**	0.181	0.307
AcRLsd	0.162	0.363	0.525

(C) 22 µm Region of Interest

Supplementary Table 3: Accuracy on Hemi-brain dataset. Tables show best network scores in bold for each RoI.

Method	VoI Split	VoI Merge	VoI Sum
Baseline	1.222	0.133	1.355
LR	1.603	0.257	1.867
Malis	0.997	0.065	1.061
FFN	1.003	**0.053**	**1.056**
MtLsd	**0.975**	0.161	1.136
AcLsd	1.222	0.189	1.411
AcRLsd	1.138	0.275	1.413

(A) Full Region of Interest

Method	VoI Split	VoI Merge	VoI Sum
Baseline	0.716	0.168	0.884
LR	1.089	0.268	1.357
Malis	0.774	0.105	0.879
FFN	0.624	**0.076**	0.700
MtLsd	0.687	0.09	0.777
AcLsd	**0.502**	0.123	**0.625**
AcRLsd	0.568	0.131	0.699

(B) Sub Region of Interest (1)

Method	VoI Split	VoI Merge	VoI Sum
Baseline	0.927	0.112	1.039
LR	1.207	0.282	1.489
Malis	0.946	**0.042**	0.988
FFN	0.701	0.079	0.780
MtLsd	0.795	0.115	0.910
AcLsd	**0.662**	0.099	**0.761**
AcRLsd	0.691	0.186	0.877

(C) Sub Region of Interest (2)

Supplementary Table 4: Accuracy on Fib-25 dataset. Tables show best network scores in bold, for each RoI.

(A) Prediction

Method	Workers	Total Process Time (seconds)	Throughput (μm^3/ GPU seconds)	teraFLOPs
LSDs	100 (2080Ti)	7,090	1.093	437,000
Baseline	60 (2080Ti)	8,449	1.528	437,000
LR	60 (2080Ti)	10,596	1.218	440,000
Malis	60 (2080Ti)	8,522	1.515	437,000
FFN	1,000 (P100)	11,054	0.07	70,500,000
MtLsd	60 (2080Ti)	9,193	1.404	440,000
AcLsd	15 (V100)	43,972	1.174	874,000
AcRLsd	24 (V100)	37,837	0.854	874,000

(B) Segmentation (MtLsd example)

Method	Workers	Total Process Time (seconds)	Throughput (μm^3/ CPU seconds)
Watershed	100 CPUs	7,780	0.996
Agglomeration	100 CPUs	19,859	0.390

Supplementary Table 5: Computational costs on Zebrafinch Benchmark Roi.

**Volume**	Baseline	LR	MtLsd	AcLsd	AcRLsd
basil_v001	0.235	0.206	**0.186**	0.210	0.445
basil_v004	0.775	0.789	0.830	**0.658**	0.979
minnie_v024	1.594	1.978	1.823	**1.440**	1.963
pinky_v104	0.244	**0.229**	0.2355	0.248	0.245
**Avg. ± SD**	0.712 ± 0.554	0.800 ± 0.719	0.769 ± 0.659	**0.639 ± 0.495**	0.908 ± 0.666

Supplementary Table 6: Best Variation of Information (Sum) per network on four testing volumes from microns data (lower is better). Best method per volume is shown in bold.

Component	MtLsd	AcLsd
Off-Diagonals of Covariance	0.1295	0.0268
Off-Diagonals of Covariance + Size	0.0332	0.0613
Full LSDs	0.0373	0.0261
Mean Offsets	0.0406	0.0344
Mean Offsets + Off-Diagonals of Covariance	0.0727	0.0457
Mean Offsets + Off-Diagonals of Covariance + Size	0.1681	0.0439
Mean Offsets + Diagonals of Covariance	0.0412	0.0299
Mean Offsets + Diagonals of Covariance + Off-Diagonals of Covariance	0.0465	**0.0256**
Mean Offsets + Diagonals of Covariance + Size	**0.0258**	0.0294
Mean Offsets + Size	0.0835	0.0749
Diagonals of Covariance	0.0491	0.0329
Diagonals of Covariance + Off-Diagonals of Covariance	0.0374	0.1168
Diagonals of Covariance + Off-Diagonals of Covariance + Size	0.0414	0.0421
Diagonals of Covariance + Size	0.0758	0.0295
Size	0.0463	0.8444

Supplementary Table 7: Best Variation of Information (Sum) per component combination for both networks (lower is better).

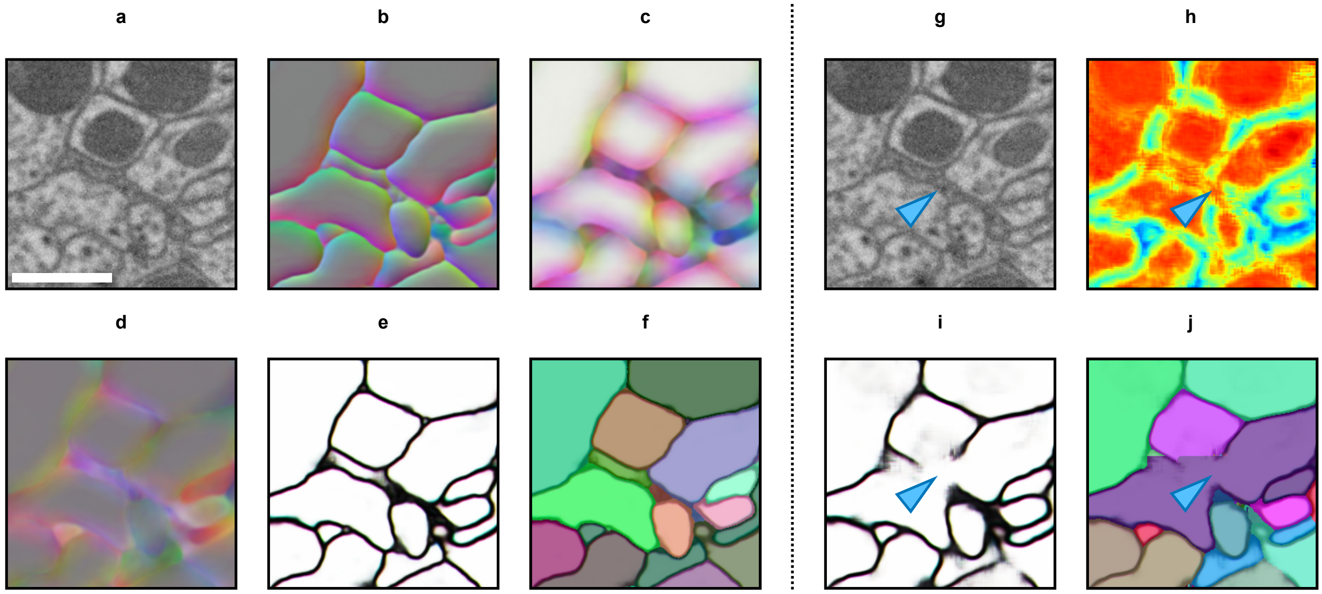

Supplementary Figure 2: Results from best autocontext ablation components (left of dashed line) and worst components (right of dashed line) on Hemi-brain cutout. **A, G**. Raw EM data, scale bar = 500 nm. **B,C,D**. Mean offset, orthogonals, and diagonals of LSDs in best ablation. Best performing ablation did not include a size component. **E, F**. Affinities and segmentation produced from best ablation. **H**. Size (and sole) component of LSDs in worst ablation. **I, J**. Affinities and segmentation produced from worst ablation. Autocontext using just the size component as input causes large gaps in the affinities resulting in a false merge (blue arrows).

Volume	LR	AcLsd
N_522	1.881	**1.388**
N_593	**1.644**	1.740
N_441	1.462	**1.196**
N_435	1.671	**1.495**
N_590	**1.474**	1.636
N_511	**1.153**	1.290
N_294	2.021	**1.604**
**Avg. ± SD**	1.615 ± 0.266	**1.478 ± 0.182**

Supplementary Table 8: Best Variation of Information (Sum) for each network over seven testing volumes (lower is better).

Parameter	Value
Input feature maps	12
Layer fmap scale	5
Downsampling factors	[[1,3,3],[1,3,3],[3,3,3]]
Output feature maps	14
Input shape	[84,268,268]
Output shape	[48,56,56]
Loss	MSE
Optimizer	Adam
Learning rate	0.5×10^{-4}
β_1	0.95
β_2	0.999
ϵ	1×10^{-8}
Iterations	400,000

Augmentation	Parameter	Value
Elastic	control point spacing	(4,4,10)
	jitter sigma	(0, 2, 2)
	subsample	8
Rotation	axis	x,y,z
	angle	in $[0, 2\pi]$
Section Defects	slip probability	0.05
	shift probability	0.05
	max misalign	10
Mirror	axes	x,y,z
Transpose	axes	x, y
Intensity	scale	in $[0.9, 1.1]$
	shift	in $[-0.1, 0.1]$

Supplementary Table 9: Training parameters and augmentations[9] of MtLsd network on Zebrafinch dataset.

Parameter	Value
Input feature maps	12
Layer fmap scale	6
Downsampling factors	[[2,2,2],[2,2,2],[3,3,3]]
Output feature maps	14
Input shape	[196,196,196]
Output shape	[92,92,92]
Loss	MSE
Optimizer	Adam
Learning rate	0.5×10^{-4}
β_1	0.95
β_2	0.999
ϵ	1×10^{-8}
Iterations	400,000

Augmentation	Parameter	Value
Elastic 1	control point spacing	(40,40,40)
	jitter sigma	(0, 0, 0)
	subsample	8
Rotation 1	axis	x,y,z
	angle	in $[0, 2\pi]$
Section Defects 1	slip probability	0
	shift probability	0
	max misalign	0
Mirror	axes	x,y,z
Transpose	axes	x,y,z
Elastic 2	control point spacing	(40,40,40)
	jitter sigma	(2, 2, 2)
	subsample	8
Rotation 2	axis	x,y,z
	angle	in $[0, 2\pi]$
Section Defects 2	slip probability	0.01
	shift probability	0.01
	max misalign	1
Intensity	scale	in $[0.9, 1.1]$
	shift	in $[-0.1, 0.1]$

Supplementary Table 10: Training parameters and augmentations[9] of MtLsd network on FIB-SEM datasets.

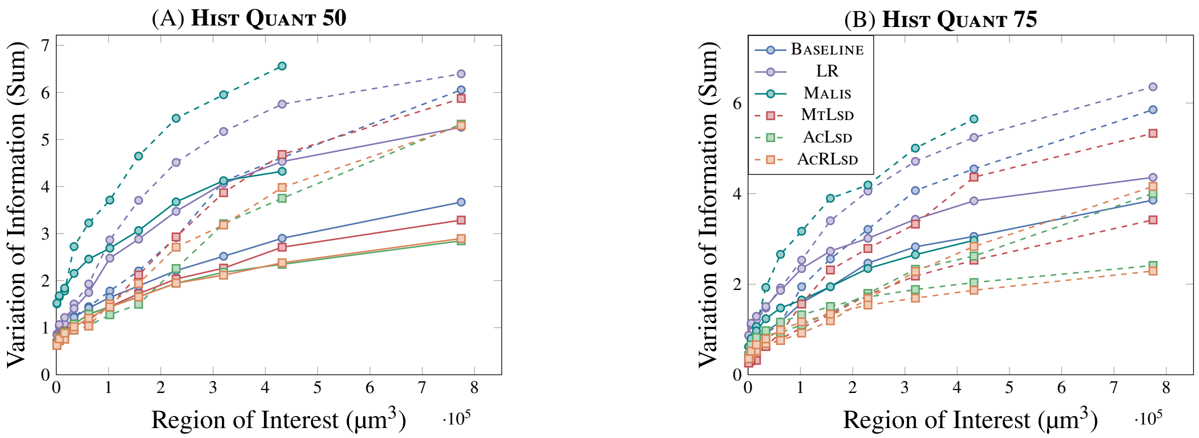

Supplementary Figure 3: VoI Sum vs RoI on masked (solid) and non-masked (dashed) Zebrafinch data.

Operation	FLOPs
unet_layer_2_left_1/convolution	940584960000
unet_layer_2_right_0/convolution	786542400000
unet_layer_3_left_1/convolution	419904000000
unet_layer_1_left_1/convolution	416320819200
unet_layer_2_right_1/convolution	338411520000
unet_layer_1_right_0/convolution	226738828800
unet_layer_2_left_0/convolution	208980000000
unet_layer_0_left_1/convolution	164826316800
unet_layer_3_left_0/convolution	123832800000
unet_layer_1_right_1/convolution	105305702400
unet_layer_1_left_0/convolution	87354028800
unet_layer_0_right_0/convolution	84455094528
gradients/unet_up_3_to_2/conv3d_transpose_grad/Conv3D	83980800000
unet_layer_0_right_1/convolution	46982799360
gradients/unet_up_2_to_1/conv3d_transpose_grad/Conv3D	22560768000
unet_layer_0_left_0/convolution	14151319488
gradients/unet_up_1_to_0/conv3d_transpose_grad/Conv3D	7020380160
embedding_0/convolution	1242931200
affs_0/convolution	372879360
unet_layer_0_left_0/BiasAdd	262061472
gradients/unet_layer_0_left_0/BiasAdd_grad/BiasAddGrad	262061460
gradients/AddN_3	254361600
unet_layer_0_left_1/BiasAdd	254361600
gradients/unet_layer_0_left_1/BiasAdd_grad/BiasAddGrad	254361588
unet_layer_1_left_0/BiasAdd	134805600
gradients/unet_layer_1_left_0/BiasAdd_grad/BiasAddGrad	134805540
unet_layer_1_left_1/BiasAdd	128494080
gradients/AddN_2	128494080
gradients/unet_layer_1_left_1/BiasAdd_grad/BiasAddGrad	128494020
unet_layer_3_left_1/kernel/Initializer/random_uniform	60750000
mean_squared_error/num_present	44390399
mean_squared_error/SquaredDifference	88780800
unet_layer_0_right_0/BiasAdd	65165968
gradients/unet_layer_0_right_0/BiasAdd_grad/BiasAddGrad	65165954
unet_layer_2_left_0/BiasAdd	64500000
gradients/unet_layer_2_left_0/BiasAdd_grad/BiasAddGrad	64499700
unet_layer_0_right_1/BiasAdd	62146560
gradients/AddN	62146560
gradients/unet_layer_0_right_1/BiasAdd_grad/BiasAddGrad	62146546
unet_up_1_to_0/BiasAdd	58503168
gradients/unet_up_1_to_0/BiasAdd_grad/BiasAddGrad	58503156
unet_layer_2_left_1/BiasAdd	58060800
gradients/AddN_1	58060800
gradients/unet_layer_2_left_1/BiasAdd_grad/BiasAddGrad	58060500
embedding_0/BiasAdd	44390400
gradients/mean_squared_error/Mul_grad/mul	44390400
gradients/mean_squared_error/Mul_grad/mul_1	44390400
gradients/mean_squared_error/SquaredDifference_grad/Neg	44390400
gradients/mean_squared_error/SquaredDifference_grad/mul	44390400
mean_squared_error/Mul	44390400
gradients/mean_squared_error/SquaredDifference_grad/mul_1	44390400
gradients/mean_squared_error/SquaredDifference_grad/sub	44390400
mean_squared_error/Sum	44390399
gradients/embedding_0/BiasAdd_grad/BiasAddGrad	44390390
gradients/mean_squared_error/Mul_grad/Sum_1	39951360
unet_up_2_to_1/BiasAdd	37601280
gradients/unet_up_2_to_1/BiasAdd_grad/BiasAddGrad	37601220
unet_layer_1_right_0/BiasAdd	34990560
gradients/unet_layer_1_right_0/BiasAdd_grad/BiasAddGrad	34990500
unet_layer_1_right_1/BiasAdd	32501760
gradients/unet_layer_1_right_1/BiasAdd_grad/BiasAddGrad	32501700
unet_up_3_to_2/BiasAdd	27993600
gradients/unet_up_3_to_2/BiasAdd_grad/BiasAddGrad	27993300
mean_squared_error_1/SquaredDifference	26634240
mean_squared_error_1/num_present	13317119
unet_layer_3_left_0/kernel/Initializer/random_uniform	12150000
unet_up_3_to_2/kernel/Initializer/random_uniform	12150000
unet_layer_2_right_0/BiasAdd	24276000
gradients/unet_layer_2_right_0/BiasAdd_grad/BiasAddGrad	24275700
unet_layer_2_right_1/BiasAdd	20889600
gradients/unet_layer_2_right_1/BiasAdd_grad/BiasAddGrad	20889300
gradients/mean_squared_error_1/SquaredDifference_grad/mul	13317120
gradients/mean_squared_error_1/SquaredDifference_grad/Neg	13317120
gradients/mean_squared_error_1/SquaredDifference_grad/mul_1	13317120
mean_squared_error_1/Mul	13317120
gradients/mean_squared_error_1/Mul_grad/mul_1	13317120
gradients/mean_squared_error_1/Mul_grad/mul	13317120
affs_0/BiasAdd	13317120
gradients/mean_squared_error_1/SquaredDifference_grad/sub	13317120
mean_squared_error_1/Sum	13317119
gradients/affs_0/BiasAdd_grad/BiasAddGrad	13317117
unet_layer_2_right_0/kernel/Initializer/random_uniform	4860000
unet_layer_3_left_0/BiasAdd	7644000
gradients/unet_layer_3_left_0/BiasAdd_grad/BiasAddGrad	7642500
unet_layer_3_left_1/BiasAdd	5184000
gradients/unet_layer_3_left_1/BiasAdd_grad/BiasAddGrad	5182500
unet_layer_2_right_1/kernel/Initializer/random_uniform	2430000
unet_layer_2_left_1/kernel/Initializer/random_uniform	2430000
unet_layer_2_left_0/kernel/Initializer/random_uniform	486000
unet_layer_1_right_0/kernel/Initializer/random_uniform	194400
unet_up_2_to_1/kernel/Initializer/random_uniform	162000
unet_layer_1_right_1/kernel/Initializer/random_uniform	97200
unet_layer_1_left_1/kernel/Initializer/random_uniform	97200
unet_layer_1_left_0/kernel/Initializer/random_uniform	19440
unet_layer_0_right_0/kernel/Initializer/random_uniform	9072
unet_up_1_to_0/kernel/Initializer/random_uniform	6480
unet_layer_0_right_1/kernel/Initializer/random_uniform	5292
unet_layer_0_left_1/kernel/Initializer/random_uniform	3888
unet_layer_0_left_0/kernel/Initializer/random_uniform	324
embedding_0/kernel/Initializer/random_uniform	140
affs_0/kernel/Initializer/random_uniform	42
gradients/Slice_1_grad/sub	5
gradients/Slice_1_grad/sub_1	5
gradients/Slice_2_grad/sub	5
gradients/Slice_2_grad/sub_1	5
gradients/Slice_grad/sub	5
gradients/Slice_grad/sub_1	5
mean_squared_error_1/Greater	1
unet_up_1_to_0/mul	1
unet_up_3_to_2/mul_2	1
unet_up_3_to_2/mul_1	1
unet_up_3_to_2/mul	1
unet_up_3_to_2/add_2	1
unet_up_3_to_2/add_1	1
unet_up_3_to_2/add	1
unet_up_2_to_1/mul_2	1
unet_up_2_to_1/mul_1	1
unet_up_2_to_1/mul	1
unet_up_2_to_1/add_2	1
unet_up_2_to_1/add_1	1
unet_up_2_to_1/add	1
unet_up_1_to_0/mul_2	1
unet_up_1_to_0/mul_1	1
gradients/mean_squared_error_1/div_grad/RealDiv_2	1
Adam/mul_1	1
add	1
gradients/mean_squared_error/div_grad/Neg	1
gradients/mean_squared_error/div_grad/RealDiv	1
gradients/mean_squared_error/div_grad/RealDiv_1	1
gradients/mean_squared_error/div_grad/RealDiv_2	1
gradients/mean_squared_error/div_grad/mul	1
gradients/mean_squared_error_1/div_grad/Neg	1
gradients/mean_squared_error_1/div_grad/RealDiv	1
gradients/mean_squared_error_1/div_grad/RealDiv_1	1
unet_up_1_to_0/add_2	1
gradients/mean_squared_error_1/div_grad/mul	1
mean_squared_error/Equal	1
mean_squared_error/Greater	1
mean_squared_error/div	1
mean_squared_error_1/Equal	1
Adam/mul	1
mean_squared_error_1/div	1
unet_up_1_to_0/add	1
unet_up_1_to_0/add_1	1
Total	4083673765073

Supplementary Table 11: FLOPs breakdown by operation for MtLsd network on Zebrafinch dataset.

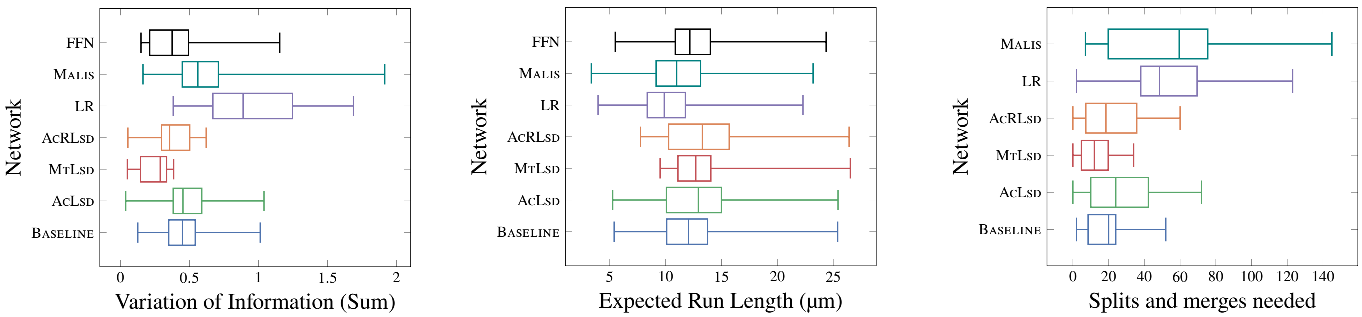

Supplementary Figure 4: Metric distribution across randomly sampled non-masked sub RoIs in the ZEBRAFINCH. N=11 RoIs. All box plots show lines at the median and quartiles.

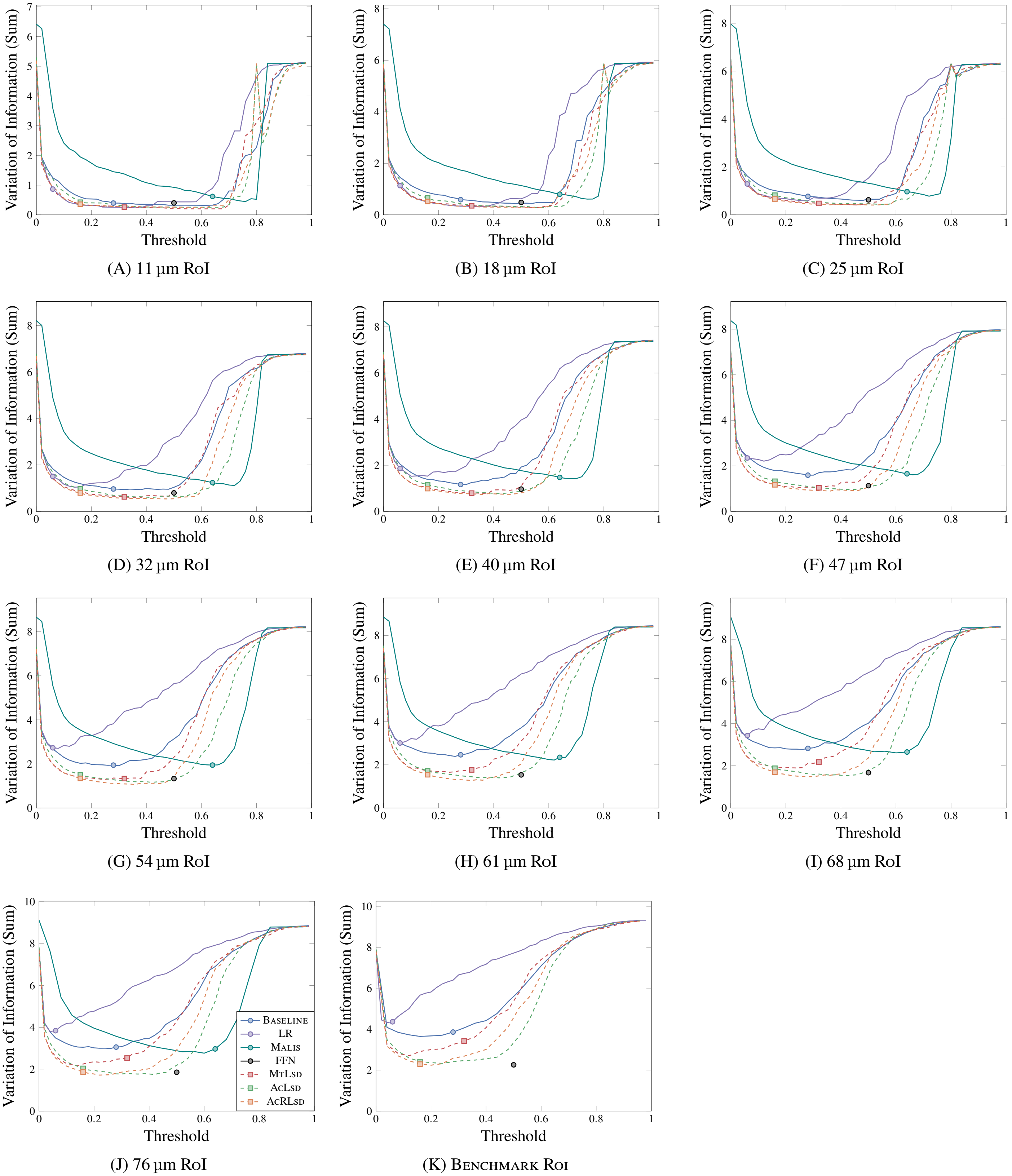

(A) 11 µm RoI (B) 18 µm RoI (C) 25 µm RoI (D) 32 µm RoI (E) 40 µm RoI (F) 47 µm RoI (G) 54 µm RoI (H) 61 µm RoI (I) 68 µm RoI (J) 76 µm RoI (K) Benchmark Roi

Supplementary Figure 5: VoI Sum vs threshold across Zebrafinch RoIs. Points correspond to thresholds which minimized VoI Sum on the validation dataset.

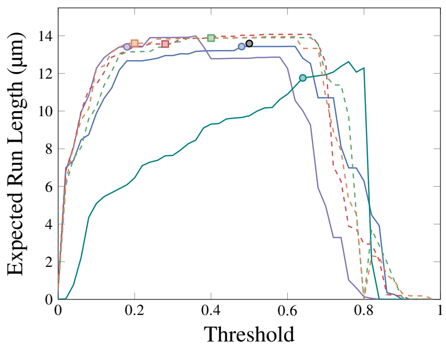

(A) 11 µm RoI

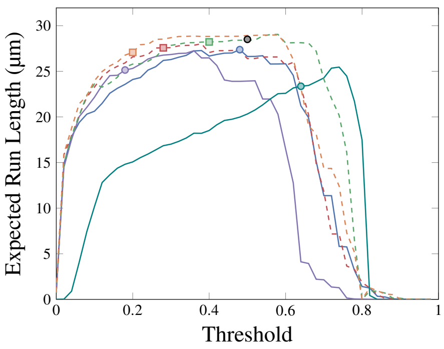

(B) 18 µm RoI

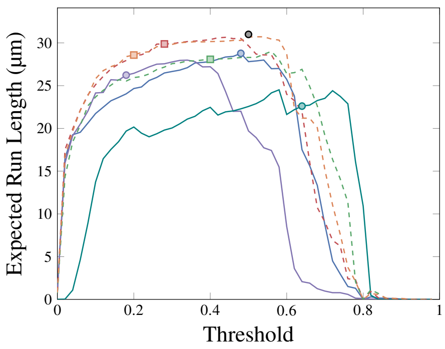

(C) 25 µm RoI

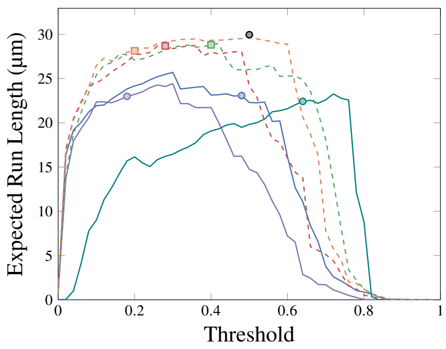

(D) 32 µm RoI

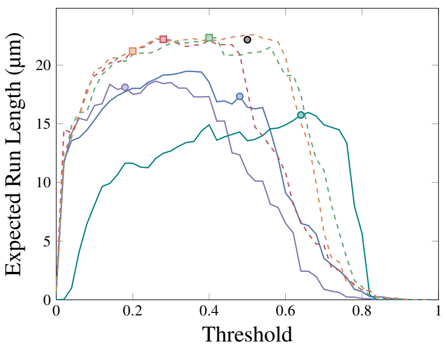

(E) 40 µm RoI

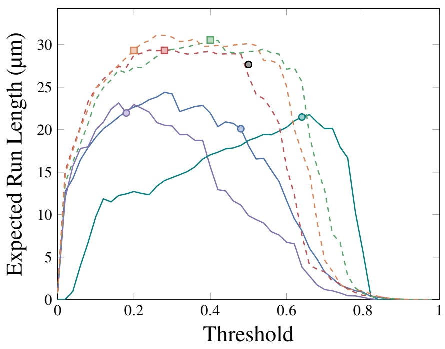

(F) 47 µm RoI

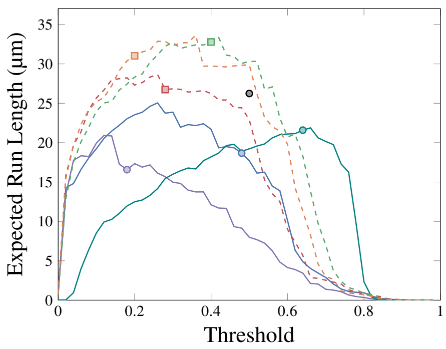

(G) 54 µm RoI

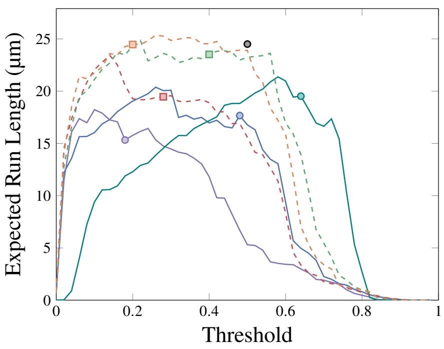

(H) 61 µm RoI

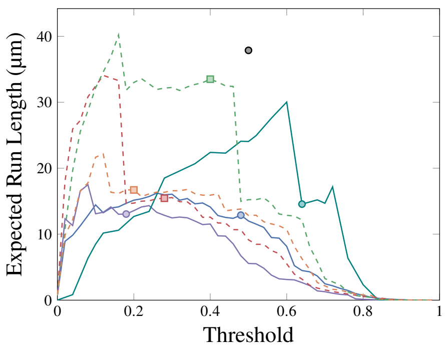

(I) 68 µm RoI

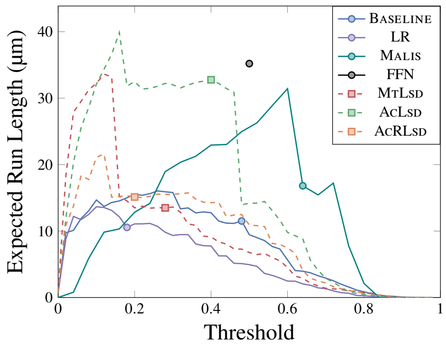

(J) 76 µm RoI

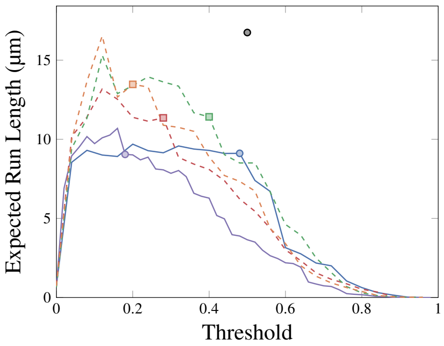

(K) BENCHMARK ROI

Supplementary Figure 6: ERL vs threshold across ZEBRAFINCH RoIs. Points correspond to thresholds which maximized ERL on the validation dataset.

Hist Quant 50

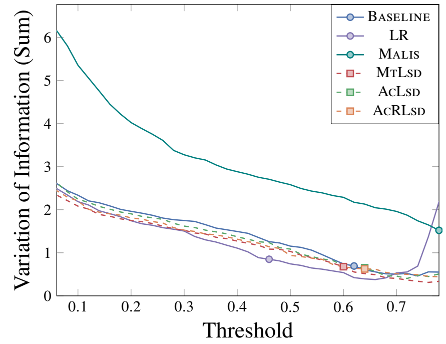

(A) VoI Sum vs threshold 11 µm RoI

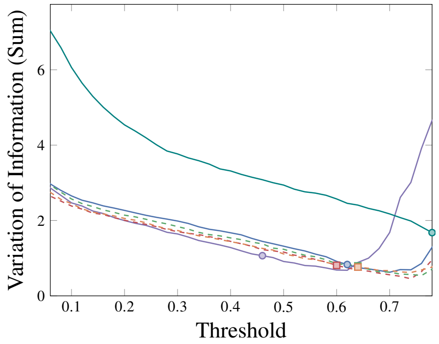

(B) VoI Sum vs threshold 18 µm RoI

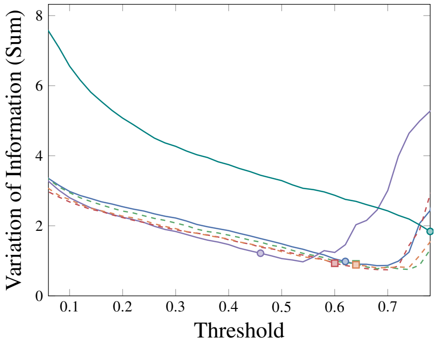

(C) VoI Sum vs threshold 25 µm RoI

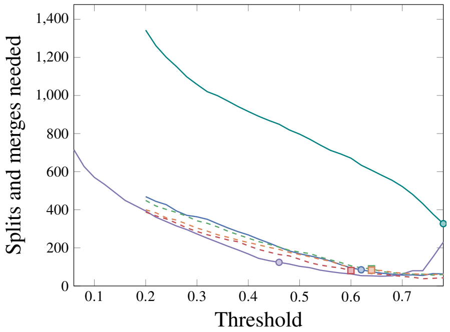

(D) MCM Sum vs threshold 11 µm RoI

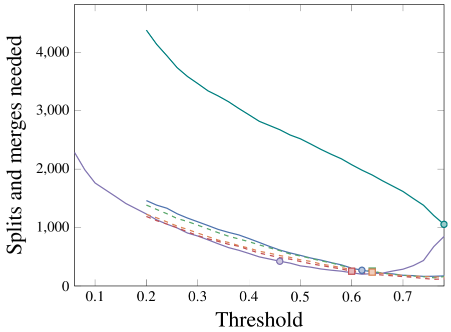

(E) MCM Sum vs threshold 18 µm RoI

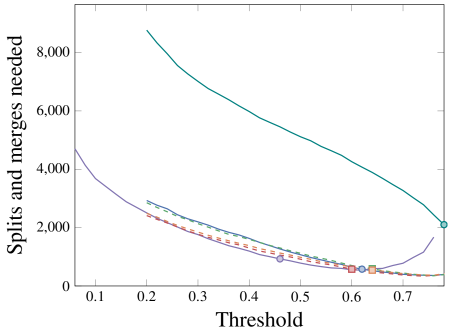

(F) MCM Sum vs threshold 25 µm RoI

Hist Quant 75

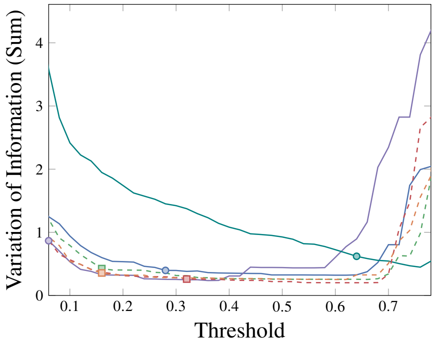

(G) VoI Sum vs threshold 11 µm RoI

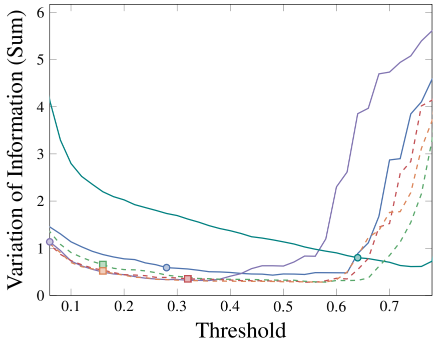

(H) VoI Sum vs threshold 18 µm RoI

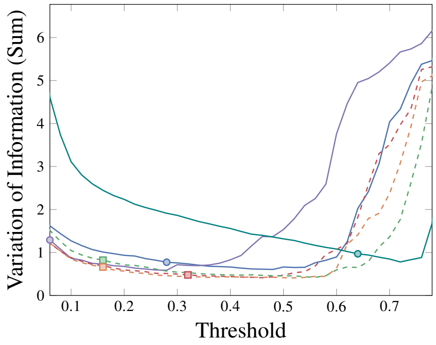

(I) VoI Sum vs threshold 25 µm RoI

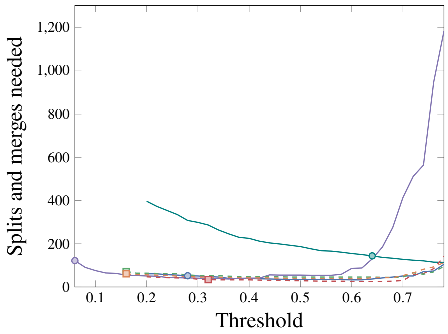

(J) MCM Sum vs threshold 11 µm RoI

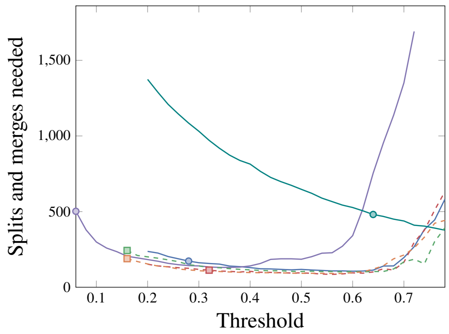

(K) MCM Sum vs threshold 18 µm RoI

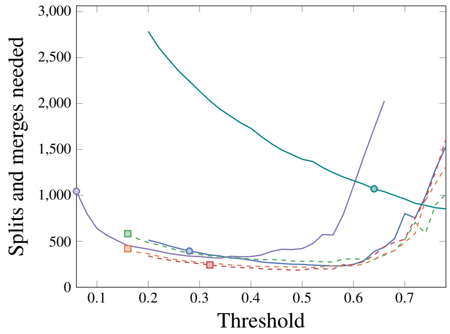

(L) MCM Sum vs threshold 25 µm RoI

Supplementary Figure 7: VoI serves as a reasonable proxy for evaluating large volumes. Comparison between VoI and MCM on Zebrafinch on first three RoIs. Similarities are consistent on both Hist Quant 50 (top two rows) and Hist Quant 75 merge functions (bottom two rows). VoI plots are cropped to the threshold range used in the MCM.

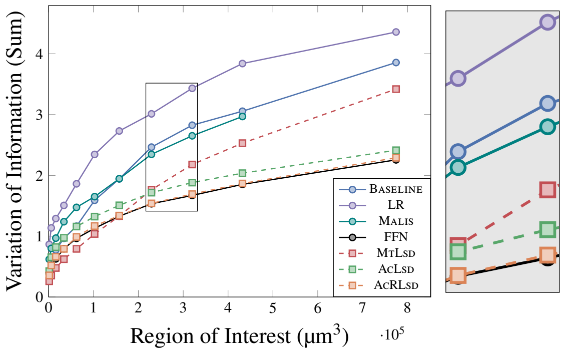

(A) RoI size vs VoI sum

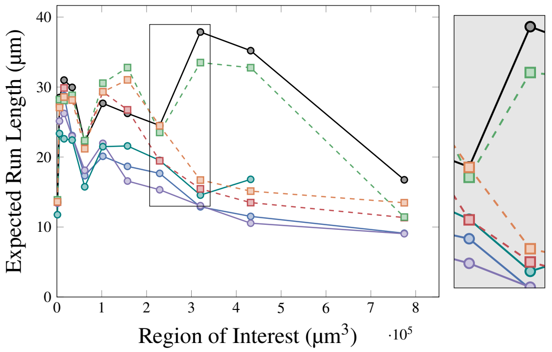

(B) RoI size vs ERL

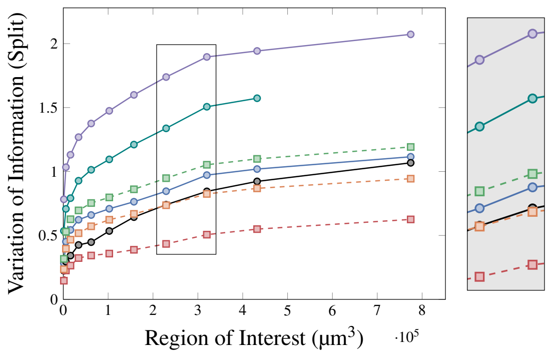

(C) RoI size vs VoI split

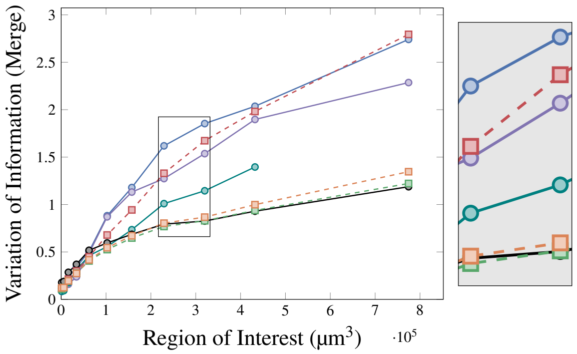

(D) RoI size vs VoI merge

Supplementary Figure 8: Demonstration of ERL sensitivity. When transitioning from a ~61μm³ to a ~68μm³ RoI, the VoI Sum increases as expected (**A**). This is not the case when considering ERL. All networks, with the exception of FFN and AcLsd, show decreases (**B**). Breaking VoI Sum into false splits (**C**.) and merges (**D**.) shows consistent increases across all networks. However, not all networks reflect this change when considering ERL. This can be best explained by the fact that ERL is more sensitive to different types of merges. In this example, AcRLsd likely merged a small fragment into a larger neuron while AcLsd likely merged two smaller fragments together. While both cases would produce a similar increase in VoI, the ERL in the former is drastically reduced. This is not reflective of the fact that both cases would require a single split to resolve in the context of a contemporary proofreading tool.

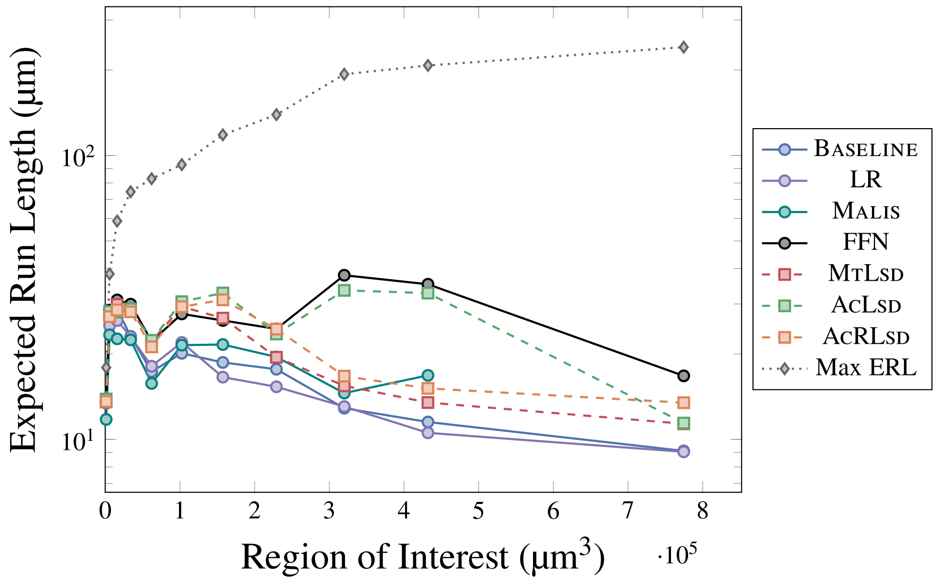

Supplementary Figure 9: RoI size vs ERL. Grey dotted line shows maximum possible ERL for each RoI. Y-axis is on a log scale

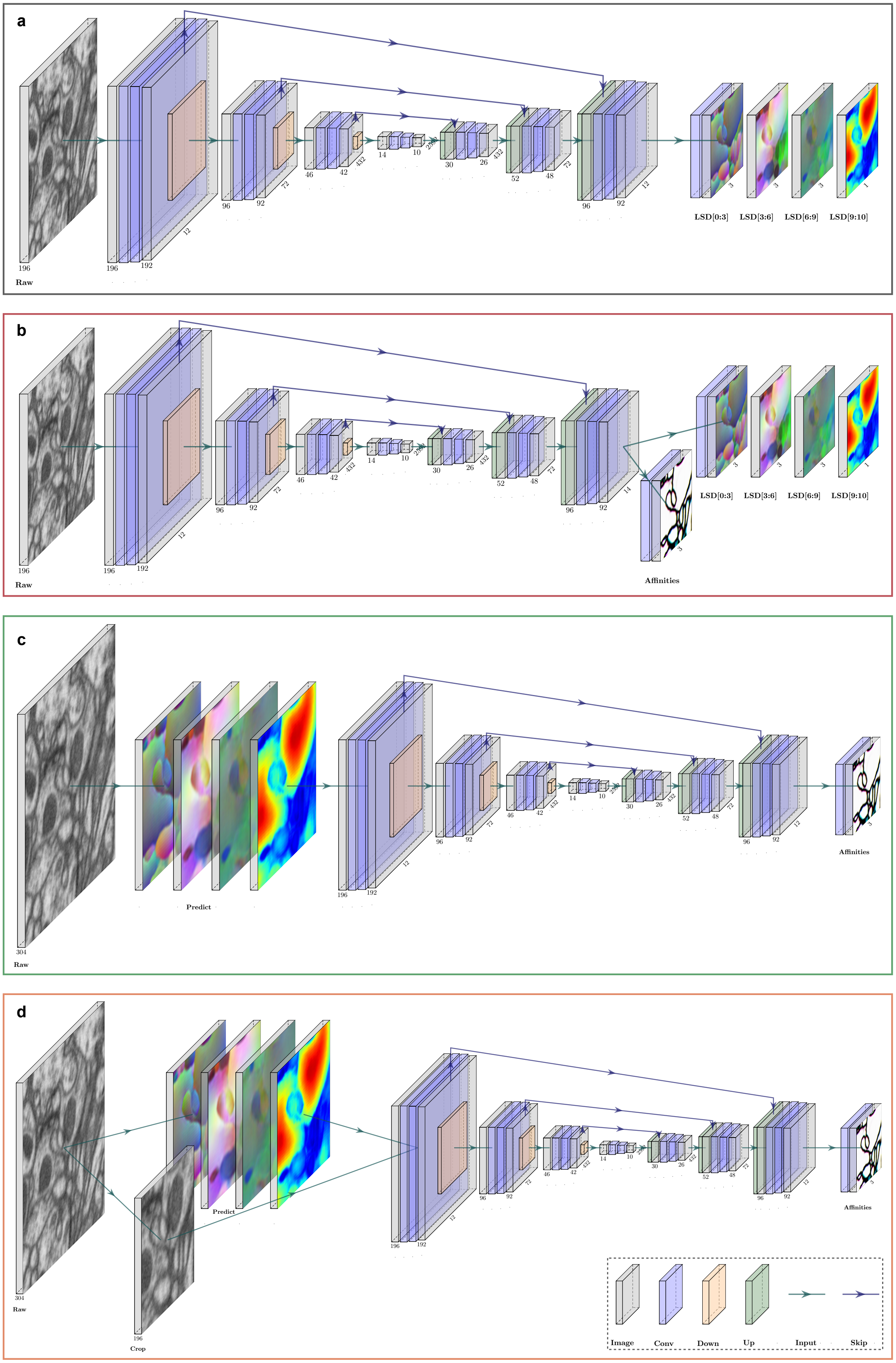

Supplementary Figure 10: Network architectures used on FIB-SEM datasets (2D representations). All architectures use the U-NET proposed in Funke et al. (2019). Legend on bottom right. **A**. Network to generate the LSDs used in auto-context setups. An extra convolution is used to get to 10 feature maps for the embedding. **B**. MTLSD network - both affinities and LSDs are learnt. Number of output feature maps is increased from 12 to 14 to account for the 13 feature maps needed for the affinities and embedding. **C**. ACLSD network - the output from **A** is used to predict embedding from raw input. The predicted embedding is then passed in to learn affinities. **D**. ACRLSD network - same as **C**, but incorporates cropped raw as input in addition to the embedding.

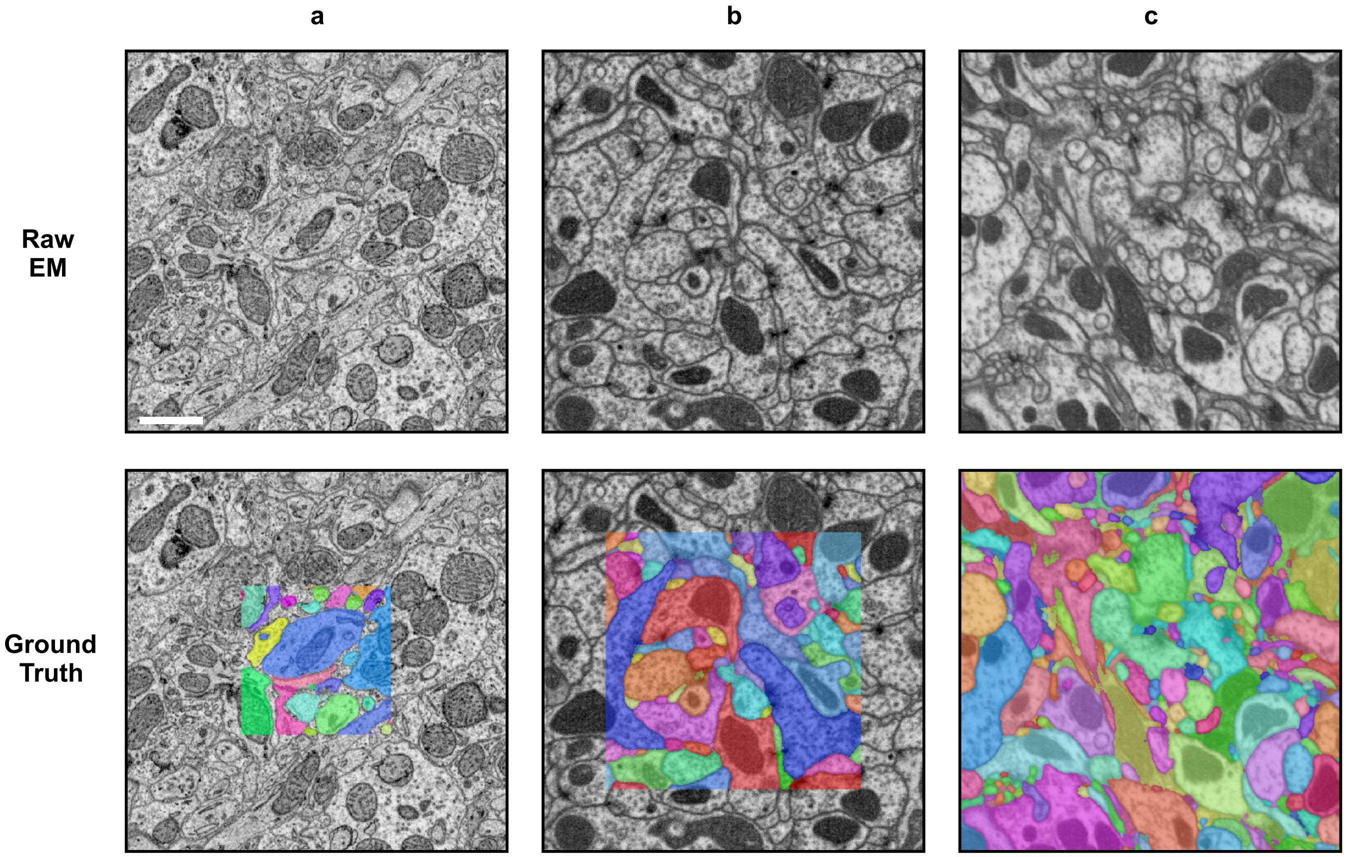

Supplementary Figure 11: Example training data. **A**. ZEBRAFINCH labels were heavily padded with raw data and some glia were set to zero (scale bar = ~ 1 µm). **B**. Padding was used to a lesser extent for HEMI-BRAIN volumes. Example taken from ELLIPSOID BODY. **C**. No padding was used for FIB-25 volumes.

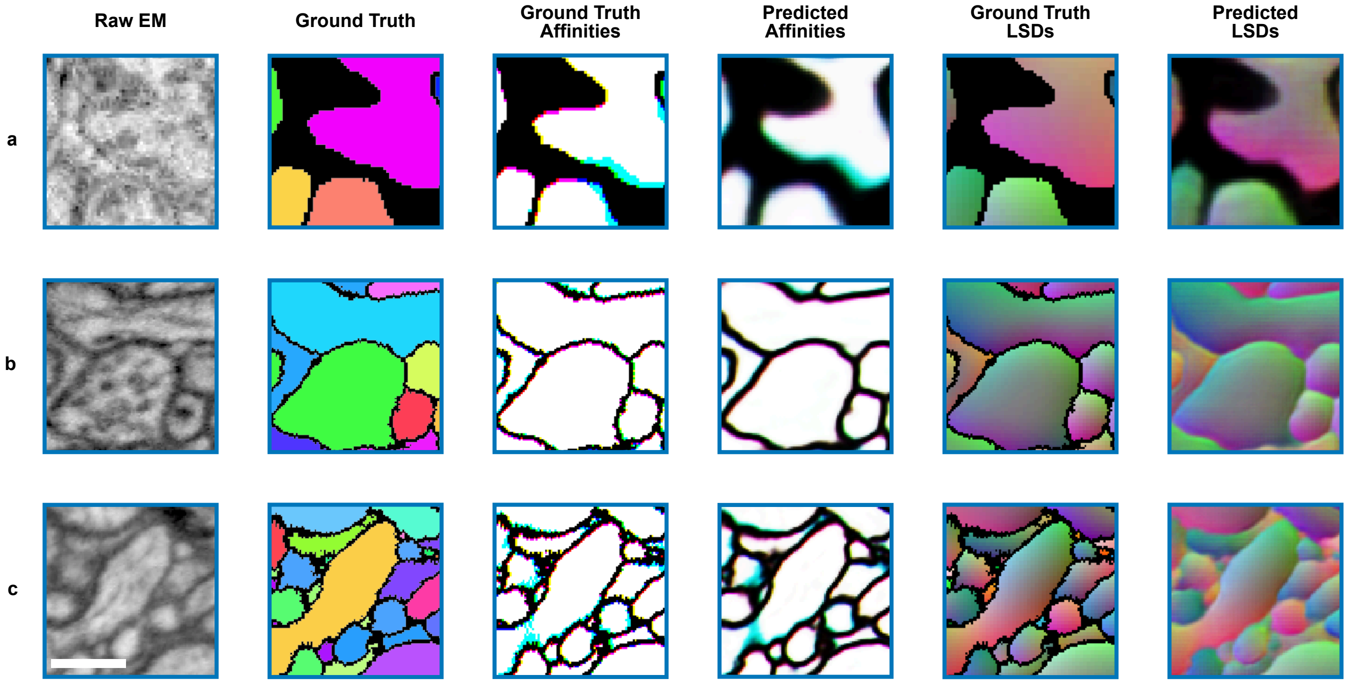

Supplementary Figure 12: Example training batches. Masked-out regions were factored into the training loss on the ZEBRAFINCH dataset (**A**). Conversely, the HEMI-BRAIN and FIB-25 volumes used no masking during training (**B,C**, respectively). Scale bar = ~300 nm.

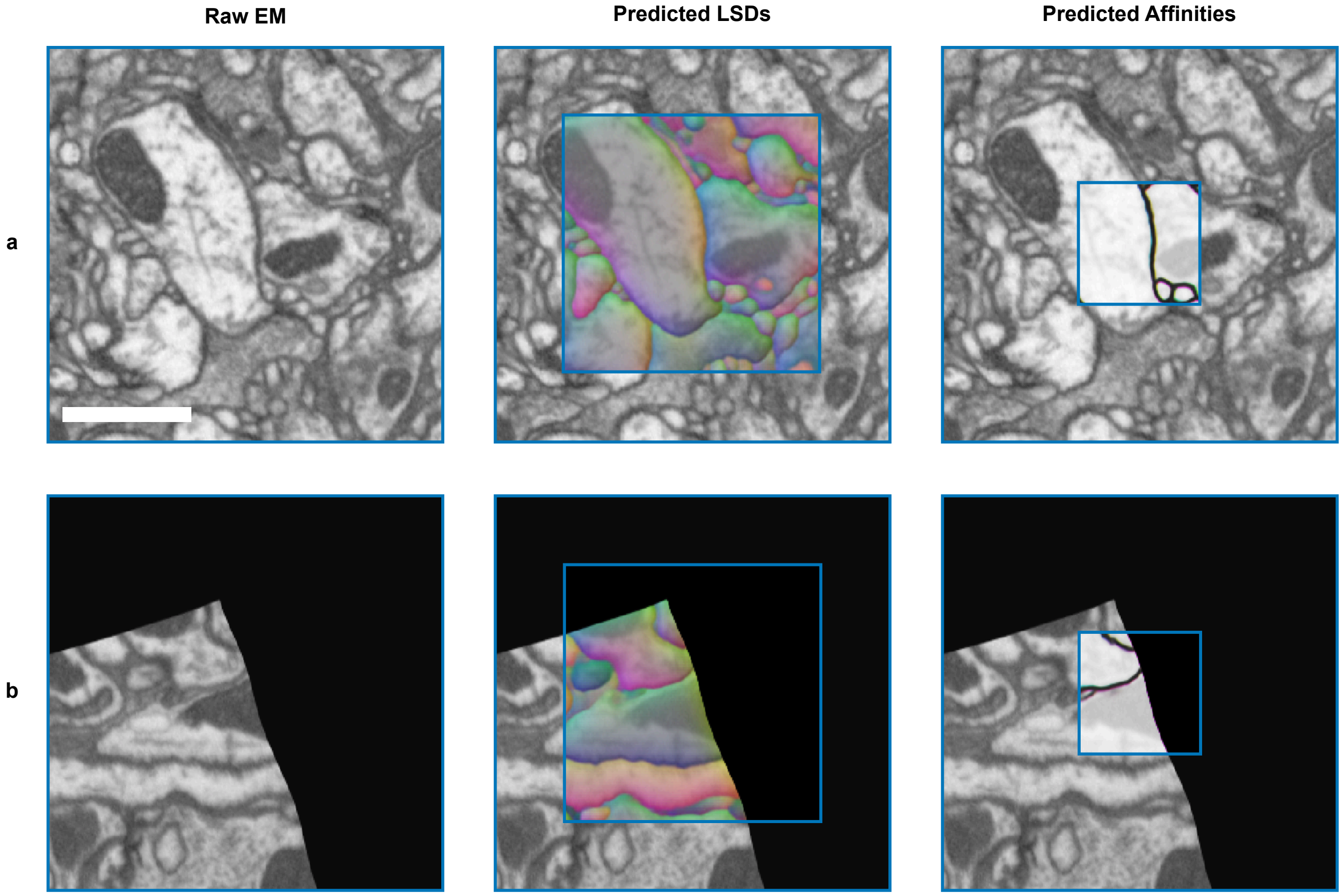

Supplementary Figure 13: Example auto-context training batches on Fib-25. **A**. Batch in which no boundary masking is needed. The first pass predicts LSDs in an intermediate RoI to provide context for affinity prediction in the second pass. **B**. Batch requires boundary masking. The combination of elastic deformation and zero padding simulates the tissue irregularities seen in the full Fib-25 volume. In these background areas, LSDs and affinities are taught to predict zero. Scale bar = ~ 1 µm.

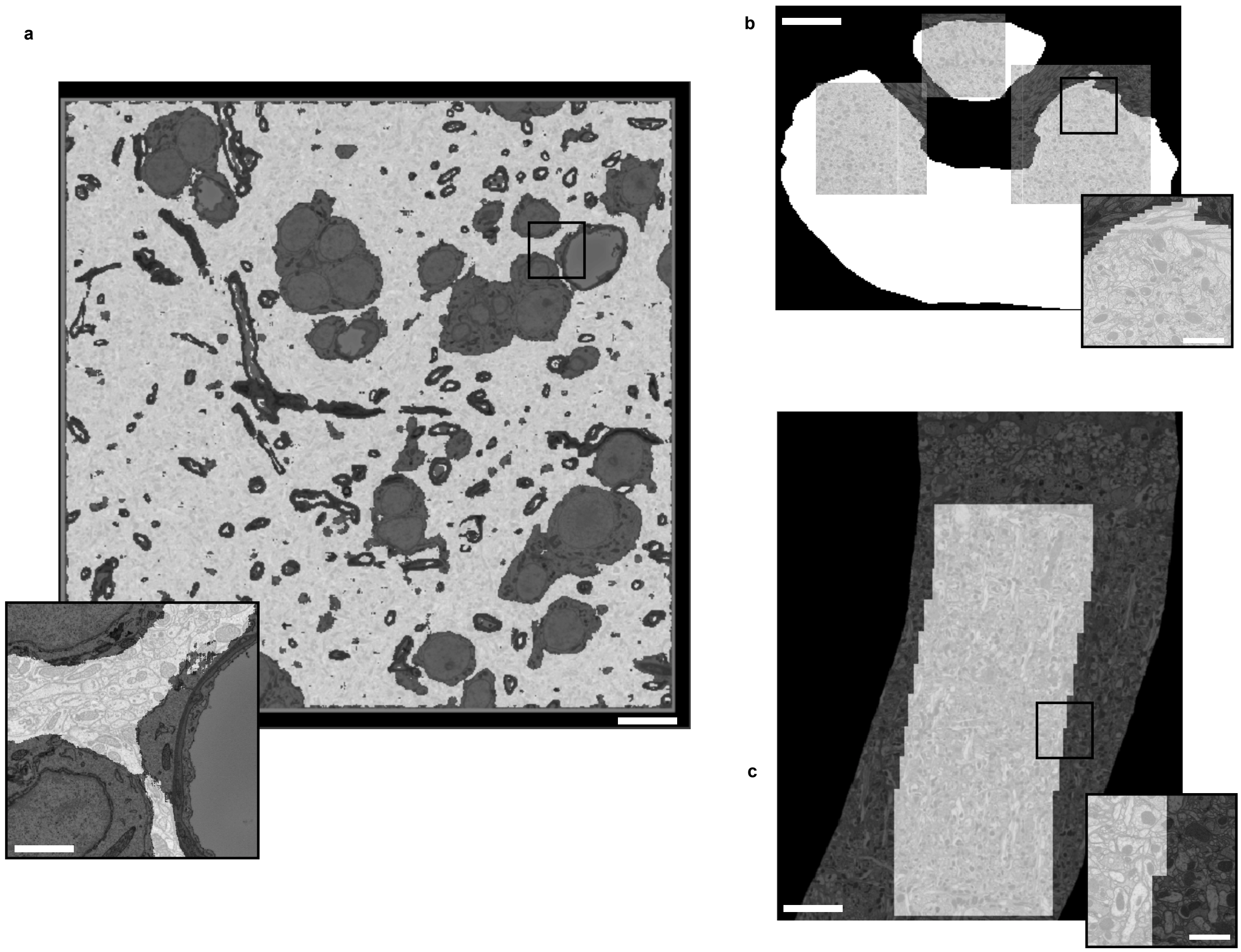

Supplementary Figure 14: Masks used in study. **A**. Zebrafinch mask removed cell bodies, myelin, blood vessels and background. Scale bar = ~10 µm. Inset scale bar = ~3 µm. **B**. Hemi-brain mask restricted volumes to Ellipsoid Body neuropil. Scale bar = ~ 10 µm. Inset scale bar = ~2 µm. **C**. Fib-25 used an irregularly shaped tissue mask, mostly limited to neuropil. Scale bar = ~8 µm. Inset scale bar = ~1 µm.

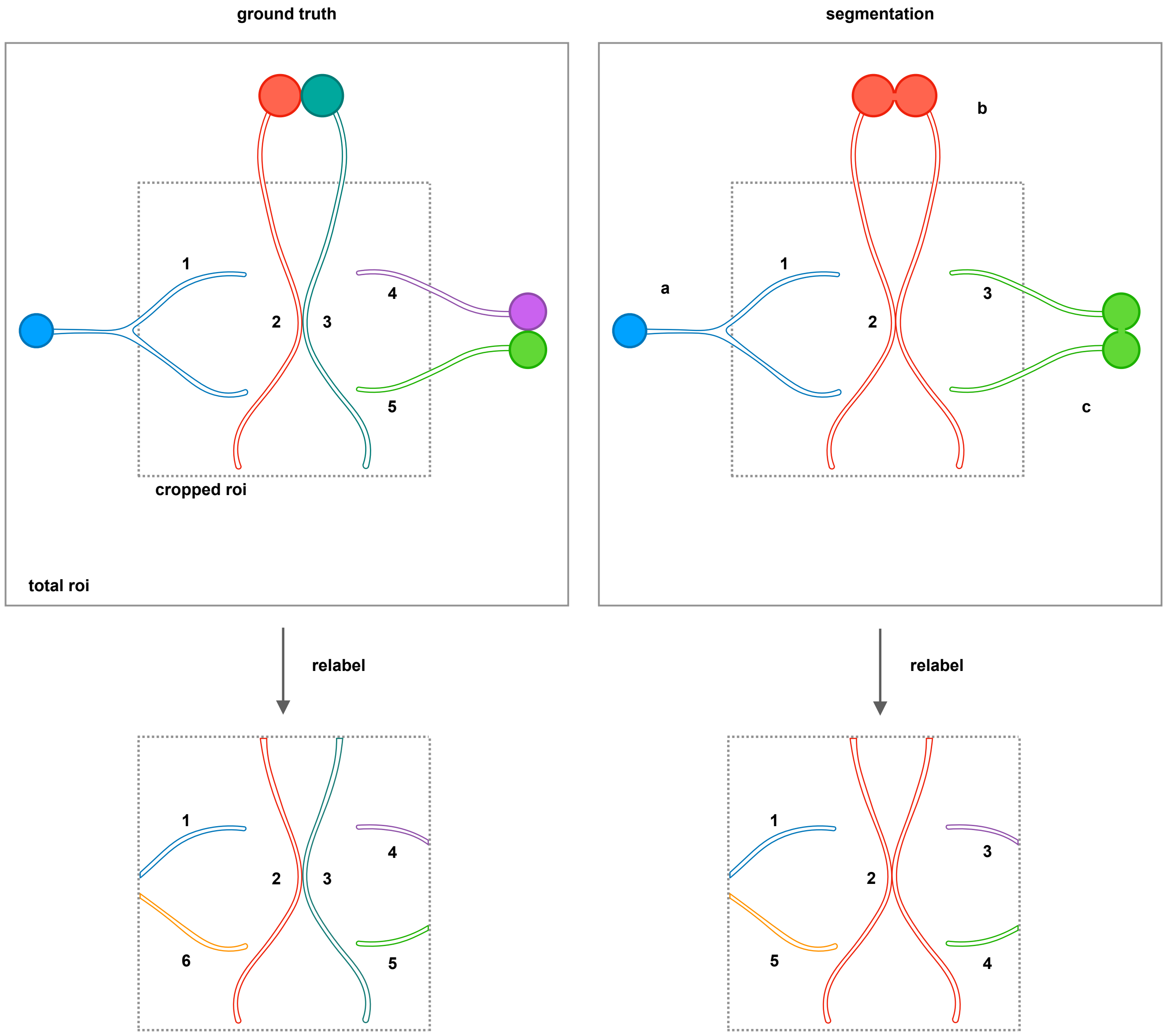

Supplementary Figure 15: Potential side effects of cropping data. Ground truth (left) shows 5 correctly labeled neurons. Example Segmentation (right) shows 3 labeled neurons as a result of false merges. Bottom squares show results from relabeling connected components inside the cropped RoI. **A**. Correctly segmented neuron in total RoI would be counted as a false merge inside cropped RoI. This is fixed by relabelling connected components and the merge/split scores are unaffected. **B**. A falsely merged neuron inside the cropped RoI is caused by a false merge outside of the cropped RoI and should not be counted. Relabelling doesnt resolve the touching boundaries and the merge score is subsequently overestimated. **C**. Incorrectly segmented neuron in total RoI is counted as correct in cropped RoI after relabelling. Merge score is underestimated.

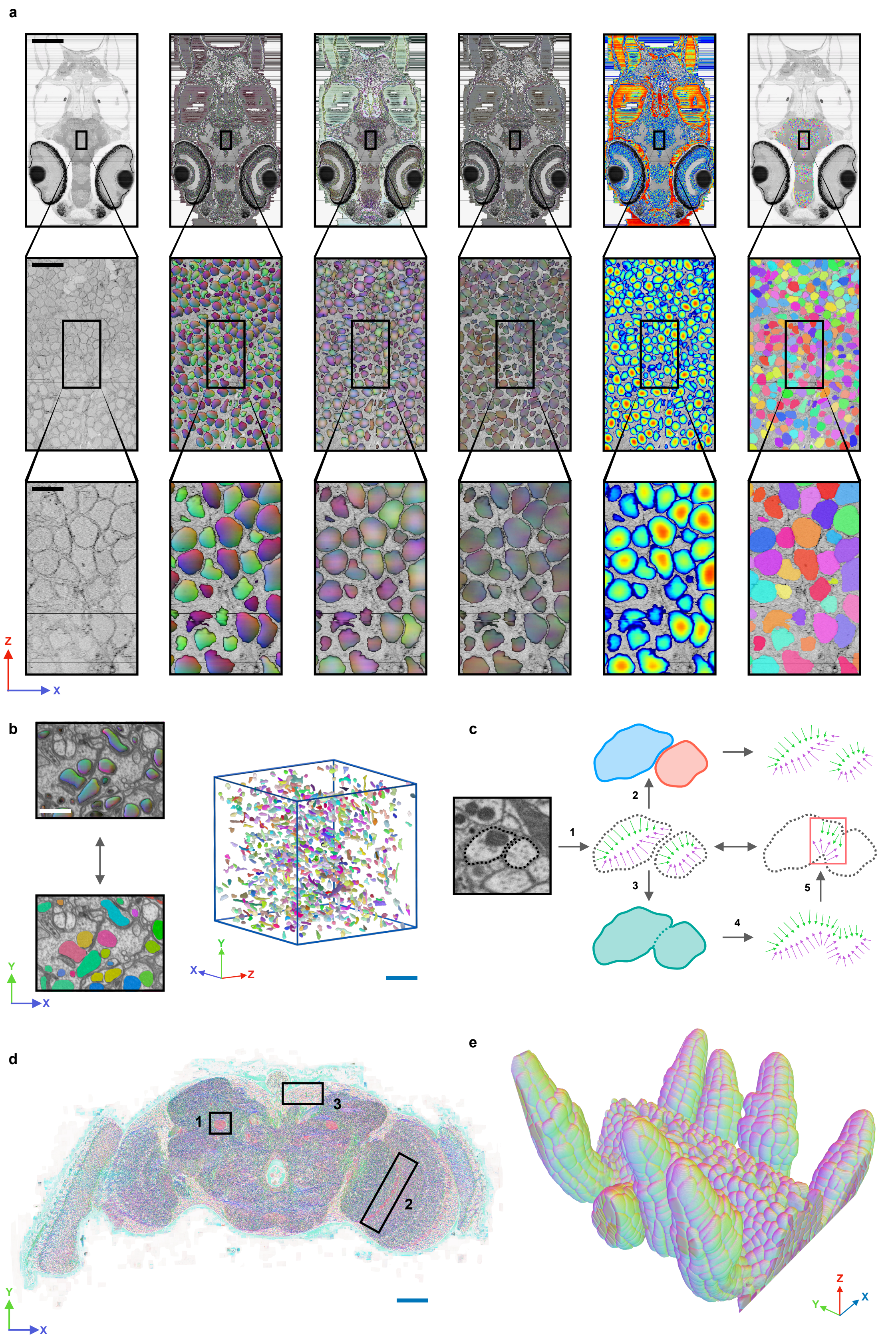

Supplementary Figure 16: Other potential uses of LSDs. **A**. Nuclei segmentation on full zebrafish brain (Hildebrand et al., 2017). Columns from left to right: raw, LSD offset vectors, LSD direction vectors (covariance), LSD direction vectors (Pearson's), size, resulting segmentation. Scale bars from top to bottom: ~ 150µm, 20µm, 5µm. **B**. Mitochondria segmentation on cropout from Fib-25. Inset shows LSD predictions and corresponding segmentation. Bottom image shows 3D reconstructions of a random sample (n=1000) in predicted RoI. Scale bars from top to bottom: ~ 3µm, 750nm, 4µm. **C**. Error mapping. Example predicted LSDs between two neurons (1). If the resulting segmentation is correct (2), segmentation LSDs do not differ from predicted LSDs. If the resulting segmentation is incorrect (3), segmentation LSDs (4) might differ from the predicted LSDs. The difference (5) could expose errors in a segmentation. **D**. Predicted direction vectors (covariance) on single section of full adult fly brain. Mushroom body pedunculi (1), optic chiasm (2), cell rind (3) highlight directionality. Scale bar = ~ 150µm. **E**. Predicted LSD mean offset component on plant epithelial cell data.

References

Beier, T., Pape, C., Rahaman, N., Prange, T., Berg, S., Bock, D. D., Cardona, A., Knott, G. W., Plaza, S. M., Scheffer, L. K., et al. (2017). Multicut brings automated neurite segmentation closer to human performance. *Nature methods*, 14(2):101–102.

Briggman, K., Denk, W., Seung, S., Helmstaedter, M., and Turaga, S. C. (2009). Maximin affinity learning of image segmentation. In *Advances in Neural Information Processing Systems*, volume 22. Curran Associates, Inc.

Ciresan, D., Giusti, A., Gambardella, L. M., and Schmidhuber, J. (2012). Deep Neural Networks Segment Neuronal Membranes in Electron Microscopy Images. In Pereira, F., Burges, C. J. C., Bottou, L., and Weinberger, K. Q., editors, *Advances in Neural Information Processing Systems 25*, pages 2843–2851. Curran Associates, Inc.

Dorkenwald, S., McKellar, C. E., Macrina, T., Kemnitz, N., Lee, K., Lu, R., Wu, J., Popovych, S., Mitchell, E., Nehoran, B., Jia, Z., Bae, J. A., Mu, S., Ih, D., Castro, M., Ogedengbe, O., Halageri, A., Kuehner, K., Sterling, A. R., Ashwood, Z., Zung, J., Brittain, D., Collman, F., Schneider-Mizell, C., Jordan, C., Silversmith, W., Baker, C., Deutsch, D., Encarnacion-Rivera, L., Kumar, S., Burke, A., Bland, D., Gager, J., Hebditch, J., Koolman, S., Moore, M., Morejohn, S., Silverman, B., Willie, K., Willie, R., Yu, S.-c., Murthy, M., and Seung, H. S. (2022). Flywire: online community for whole-brain connectomics. *Nature Methods*, 19(1):119–128.

Funke, J., Klein, J., Moreno-Noguer, F., Cardona, A., and Cook, M. (2017). TED: A Tolerant Edit Distance for segmentation evaluation. *Methods*, 115:119–127.

Funke, J., Tschopp, F., Grisaitis, W., Sheridan, A., Singh, C., Saalfeld, S., and Turaga, S. C. (2019). Large Scale Image Segmentation with Structured Loss Based Deep Learning for Connectome Reconstruction. *IEEE Transactions on Pattern Analysis and Machine Intelligence*, 41(7):1669–1680.

Hildebrand, D. G. C., Cicconet, M., Torres, R. M., Choi, W., Quan, T. M., Moon, J., Wetzel, A. W., Scott Champion, A., Graham, B. J., Randlett, O., Plummer, G. S., Portugues, R., Bianco, I. H., Saalfeld, S., Baden, A. D., Lillaney, K., Burns, R., Vogelstein, J. T., Schier, A. F., Lee, W.-C. A., Jeong, W.-K., Lichtman, J. W., and Engert, F. (2017). Whole-brain serial-section electron microscopy in larval zebrafish. *Nature*, 545(7654):345–349. Number: 7654 Publisher: Nature Publishing Group.

Januszewski, M., Kornfeld, J., Li, P. H., Pope, A., Blakely, T., Lindsey, L., Maitin-Shepard, J., Tyka, M., Denk, W., and Jain, V. (2018). High-precision automated reconstruction of neurons with flood-filling networks. *Nature Methods*, 15(8):605.

Lee, K., Lu, R., Luther, K., and Seung, H. S. (2021). Learning and Segmenting Dense Voxel Embeddings for 3D Neuron Reconstruction. *IEEE Transactions on Medical Imaging*, 40(12):3801–3811. Conference Name: IEEE Transactions on Medical Imaging.

Lee, K., Zung, J., Li, P., Jain, V., and Seung, H. S. (2017). Superhuman Accuracy on the SNEMI3d Connectomics Challenge. *arXiv:1706.00120 [cs]*.

Meilă, M. (2007). Comparing clusterings—an information based distance. *Journal of Multivariate Analysis*, 98(5):873–895.

Microns Consortium, ., Bae, J. A., Baptiste, M., Bodor, A. L., Brittain, D., Buchanan, J., Bumbarger, D. J., Castro, M. A., Celii, B., Cobos, E., Collman, F., Costa, N. M. d., Dorkenwald, S., Elabbady, L., Fahey, P. G., Fliss, T., Froudarakis, E., Gager, J., Gamlin, C., Halageri, A., Hebditch, J., Jia, Z., Jordan, C., Kapner, D., Kemnitz, N., Kinn, S., Koolman, S., Kuehner, K., Lee, K., Li, K., Lu, R., Macrina, T., Mahalingam, G., McReynolds, S., Miranda, E., Mitchell, E., Mondal, S. S., Moore, M., Mu, S., Muhammad, T., Nehoran, B., Ogedengbe, O., Papadopoulos, C., Papadopoulos, S., Patel, S., Pitkow, X., Popovych, S., Ramos, A., Reid, R. C., Reimer, J., Schneider-Mizell, C. M., Seung, H. S., Silverman, B., Silversmith, W., Sterling, A., Sinz, F. H., Smith, C. L., Suckow, S., Takeno, M., Tan, Z. H., Tolias, A. S., Torres, R., Turner, N. L., Walker, E. Y., Wang, T., Williams, G., Williams, S., Willie, K., Willie, R., Wong, W., Wu, J., Xu, C., Yang, R., Yatsenko, D., Ye, F., Yin, W., and Yu, S.-c. (2021). Functional connectomics spanning multiple areas of mouse visual cortex. Technical report, bioRxiv. Section: New Results Type: article.

Nguyen, T., Malin-Mayor, C., Patton, W., and Funke, J. (2022). Daisy: block-wise task dependencies for luigi.

Plaza, S. M. (2016). Focused Proofreading to Reconstruct Neural Connectomes from EM Images at Scale. In Carneiro, G., Mateus, D., Peter, L., Bradley, A., Tavares, J. M. R. S., Belagiannis, V., Papa, J. P., Nascimento, J. C., Loog, M., Lu, Z., Cardoso, J. S., and Cornebise, J., editors, *Deep Learning and Data Labeling for Medical Applications*, Lecture Notes in Computer Science, pages 249–258, Cham. Springer International Publishing.

Plaza, S. M. and Funke, J. (2018). Analyzing Image Segmentation for Connectomics. *Frontiers in Neural Circuits*, 12.

Scheffer, L. K., Xu, C. S., Januszewski, M., Lu, Z., Takemura, S.-y., Hayworth, K. J., Huang, G. B., Shinomiya, K., Maitlin-Shepard, J., Berg, S., Clements, J., Hubbard, P. M., Katz, W. T., Umayam, L., Zhao, T., Ackerman, D., Blakely, T., Bogovic, J., Dolafi, T., Kainmueller, D., Kawase, T., Khairy, K. A., Leavitt, L., Li, P. H., Lindsey, L., Neubarth, N., Olbris, D. J., Otsuna, H., Trautman, E. T., Ito, M., Bates, A. S., Goldammer, J., Wolff, T., Svirskas, R., Schlegel, P., Neace, E., Knecht, C. J., Alvarado, C. X., Bailey, D. A., Ballinger, S., Borycz, J. A., Canino, B. S., Cheatham, N., Cook, M., Dreher, M., Duclos, O., Eubanks, B., Fairbanks, K., Finley, S., Forknall, N., Francis, A., Hopkins, G. P., Joyce, E. M., Kim, S., Kirk, N. A., Kovalyak, J., Lauchie, S. A., Lohff, A., Maldonado, C., Manley, E. A., McLin, S., Mooney, C., Ndama, M., Ogundeyi, O., Okeoma, N., Ordish, C., Padilla, N., Patrick, C. M., Paterson, T., Phillips, E. E., Phillips, E. M., Rampally, N., Ribeiro, C., Robertson, M. K., Rymer, J. T., Ryan, S. M., Sammons, M., Scott, A. K., Scott, A. L., Shinomiya, A., Smith, C., Smith, K., Smith, N. L., Sobeski, M. A., Suleiman, A., Swift, J., Takemura, S., Talebi, I., Tarnogorska, D., Tenshaw, E., Tokhi, T., Walsh, J. J., Yang, T., Horne, J. A., Li, F., Parekh, R., Rivlin, P. K., Jayaraman, V., Costa, M., Jefferis, G. S., Ito, K., Saalfeld, S., George, R., Meinertzhagen, I. A., Rubin, G. M., Hess, H. F., Jain, V., and Plaza, S. M. (2020). A connectome and analysis of the adult Drosophila central brain. *eLife*, 9:e57443. Publisher: eLife Sciences Publications, Ltd.

Takemura, S.-y., Xu, C. S., Lu, Z., Rivlin, P. K., Parag, T., Olbris, D. J., Plaza, S., Zhao, T., Katz, W. T., Umayam, L., Weaver, C., Hess, H. F., Horne, J. A., Nunez-Iglesias, J., Aniceto, R., Chang, L.-A., Lauchie, S., Nasca, A., Ogundeyi, O., Sigmund, C., Takemura, S., Tran, J., Langille, C., Le Lacheur, K., McLin, S., Shinomiya, A., Chklovskii, D. B., Meinertzhagen, I. A., and Scheffer, L. K. (2015). Synaptic circuits and their variations within different columns in the visual system of Drosophila. *Proceedings of the National Academy of Sciences of the United States of America*, 112(44):13711–13716.

Tu, Z. and Bai, X. (2010). Auto-Context and Its Application to High-Level Vision Tasks and 3D Brain Image Segmentation. *IEEE Transactions on Pattern Analysis and Machine Intelligence*, 32(10):1744–1757. Conference Name: IEEE Transactions on Pattern Analysis and Machine Intelligence.

Turaga, S. C., Murray, J. F., Jain, V., Roth, F., Helmstaedter, M., Briggman, K., Denk, W., and Seung, H. S. (2010). Convolutional Networks Can Learn to Generate Affinity Graphs for Image Segmentation. *Neural Computation*, 22(2):511–538. Conference Name: Neural Computation.

Turner-Evans, D. B. and Jayaraman, V. (2016). The insect central complex. *Current Biology*, 26(11):R453–R457.

Wolf, S., Pape, C., Bailoni, A., Rahaman, N., Kreshuk, A., Köthe, U., and Hamprecht, F. A. (2018). The Mutex Watershed: Efficient, Parameter-Free Image Partitioning. In Ferrari, V., Hebert, M., Sminchisescu, C., and Weiss, Y., editors, *Computer Vision – ECCV 2018*, volume 11208, pages 571–587. Springer International Publishing, Cham. Series Title: Lecture Notes in Computer Science.

Wolny, A., Cerrone, L., Vijayan, A., Tofanelli, R., Barro, A. V., Louveaux, M., Wenzl, C., Strauss, S., Wilson-Sánchez, D., Lymbouridou, R., Steigleder, S. S., Pape, C., Bailoni, A., Duran-Nebreda, S., Bassel, G. W., Lohmann, J. U., Tsiantis, M., Hamprecht, F. A., Schneitz, K., Maizel, A., and Kreshuk, A. (2020). Accurate and versatile 3D segmentation of plant tissues at cellular resolution.

eLife, 9:e57613. Publisher: eLife Sciences Publications, Ltd.

Zhao, T., Olbris, D. J., Yu, Y., and Plaza, S. M. (2018). NeuTu: Software for Collaborative, Large-Scale, Segmentation-Based Connectome Reconstruction. *Frontiers in Neural Circuits*, 12.
